# Hybrid Sequencing of Full-Length cDNA Transcripts of the Medicinal Plant *Scutellaria baicalensis*

**DOI:** 10.3390/ijms20184426

**Published:** 2019-09-09

**Authors:** Ting Gao, Zhichao Xu, Xiaojun Song, Kai Huang, Ying Li, Jianhe Wei, Xunzhi Zhu, Hongwei Ren, Chao Sun

**Affiliations:** 1 Key Laboratory of Plant Biotechnology in Universities of Shandong Province, College of Life Sciences, Qingdao Agricultural University, Qingdao 266109, China (T.G.) (X.S.) (H.R.); 2 Key Lab of Chinese Medicine Resources Conservation, State Administration of Traditional Chinese Medicine of the People’s Republic of China, Institute of Medicinal Plant Development, Peking Union Medical College & Chinese Academy of Medical Sciences, Beijing 100193, China (Z.X.) (Y.L.) (J.W.); 3 Beijing igeneCode Biotech Co., Ltd, Changping District Xisanqi Center for the Olympic Century, Beijing 100096, China; 4 Institute of Botany, Jiangsu Province and Chinese Academy of Sciences, Nanjing 210014, China

**Keywords:** *Scutellaria baicalensis*, single-molecule real-time sequence, flavonoid, key genes, alternative splicing

## Abstract

*Scutellaria baicalensis* is a well-known medicinal plant that produces biologically active flavonoids, such as baicalin, baicalein, and wogonin. Pharmacological studies have shown that these compounds have anti-inflammatory, anti-bacterial, and anti-cancer activities. Therefore, it is of great significance to investigate the genetic information of *S. baicalensis,* particularly the genes related to the biosynthetic pathways of these compounds. Here, we constructed the full-length transcriptome of *S. baicalensis* using a hybrid sequencing strategy and acquired 338,136 full-length sequences, accounting for 93.3% of the total reads. After the removal of redundancy and correction with Illumina short reads, 75,785 nonredundant transcripts were generated, among which approximately 98% were annotated with significant hits in the protein databases, and 11,135 sequences were classified as lncRNAs. Differentially expressed gene (DEG) analysis showed that most of the genes related to flavonoid biosynthesis were highly expressed in the roots, consistent with previous reports that the flavonoids were mainly synthesized and accumulated in the roots of *S. baicalensis*. By constructing unique transcription models, a total of 44,071 alternative splicing (AS) events were identified, with intron retention (IR) accounting for the highest proportion (44.5%). A total of 94 AS events were present in five key genes related to flavonoid biosynthesis, suggesting that AS may play important roles in the regulation of flavonoid biosynthesis in *S. baicalensis*. This study provided a large number of highly accurate full-length transcripts, which represents a valuable genetic resource for further research of the molecular biology of *S. baicalensis*, such as the development, breeding, and biosynthesis of active ingredients.

## 1. Introduction

*Scutellaria baicalensis* Georgi (golden herb) is a medicinal plant of the Labiatae family and has been cultivated and used worldwide [1]. Its dry root, called “Huangqin”, is a staple medicinal plant product in China that has been used as an important ingredient in traditional Chinese medicine for 2000 years. As recorded in the Chinese Pharmacopoeia, Huangqin is “cold in nature” and “bitter in taste”; thus, it is a critical heat-clearing, damp-drying, fire-purging, and detoxifying drug. Huangqin has been used as a drug in dozens of countries, including Japan, Korea, and the United Kingdom. In addition to its use in Chinese medicine formulations, Huangqin has also been used as a raw material for Chinese patent medicines. The main biologically active components of *S. baicalensis* are flavonoids such as baicalin, baicalein, and wogonin, which can induce apoptosis in cancer cells without affecting normal cells [2]. Huangqin also has anti-inflammatory, antibacterial, anti-viral, anti-tumor, and hepatoprotective effects [3,4,5,6,7]. It plays a vital role in clinical medicine. However, the natural availability of *S. baicalensis* has significantly decreased over the years [8], and according to the Regulation for the Protection and Management of Wild Medicinal Resources in China, *S. baicalensis* is a third-grade endangered plant species. Its active ingredient source is also becoming increasingly limited. *S. baicalensis* has a long growth cycle in cultivation, which, coupled with other causes such as environmental and pesticide pollution, has made it very difficult for the supply of flavonoids to meet the demand of clinical application in both quantity and quality. Overall, *S. baicalensis* has great medicinal and economic value, and research on this plant is on the rise [9,10].

In recent years, with the development of high-throughput sequencing technology, transcriptome sequencing has become the main method for studying gene expression regulation. However, traditional second-generation sequencing (SGS) technology also faces some challenges, such as short read lengths and uneven exon representation due to amplification bias or tandem expression of multiple transcript isoforms [11]. Moreover, in eukaryotes, most genes are alternatively spliced, producing multiple transcripts, which greatly increases the protein-coding potential of the genome [12]. The functions of protein variants alternatively spliced from the same gene may be diverse and sometimes even opposing. The high speed, long read lengths and PCR-free methods of third-generation sequencing (TGS), such as PacBio single-molecule real-time sequencing (SMRT), enable this technology to overcome the shortcomings of traditional SGS, such as its short read lengths and incomplete coverage for the transcripts [13]. The average length of the reads in TGS is 10–15 kb, which, in combination with multifragment library screening technology, can directly yield full-length transcripts without the need for assembly, thus ensuring the accuracy of mRNA sequences and providing a new technique for full-length transcriptome profiling sequencing as well as the identification of alternative splicing (AS) isoforms [14]. In other medicinal plants, such as *Salvia miltiorrhiza, Astragalus membranaceus,* and *Caulis Dendrobii* [15,16,17], third-generation transcriptome sequencing analysis has already been performed. Notably, however, the accuracy of third-generation transcriptome sequencing is low, mostly because of deletions and insertions [13]. This situation can be improved through hybrid sequencing that combines the high-quality short reads of SGS and the low-quality long reads of TGS [18].

AS is a process in which mRNA precursors encoded by the same gene are processed through various splicing events at different sites and are further translated into diverse final protein products that exhibit distinct or mutually antagonistic functions and structural traits or that cause various phenotypes due to differences in expression levels in the same cell [19,20]. AS can be divided into seven categories: alternative 3’ splice site (A3), alternative 5’ splice site (A5), intron retention (IR), alternative first exon (AF), alternative last exon (AL), skipping exon (SE), and mutual exon exclusion (MX) [21,22,23]. AS can lead to the loss or acquisition of functional domains of a protein or premature termination of transcription, thereby affecting the function of the protein. Therefore, it causes multiple effects in gene and protein expression regulation and introduces complexity and diversity into eukaryotic transcriptomes. To understand plant development and gene function, elucidating the mechanisms of the selective splicing of genes is important. However, due to limitations in the research techniques and tools for transcriptome analysis, AS is still poorly understood. The long read length afforded by the SMRT technique provides a good tool for accurately studying AS events. With regard to the flavonoid biosynthesis pathway of the medicinal plant *S. baicalensis*, a variety of questions, e.g., whether various AS modes are present in key genes and which splicing modes lead to higher activity and better function of the ingredients, and directly regulate flavonoid synthesis, are worth studying.

In this study, we cost-effectively acquired the full-length transcriptome of *S. baicalensis* via hybrid sequencing technologies, constructed a unigene library, and explored the reference sequences of this species at the transcript level. On this basis, we systematically conducted functional annotation of the full-length transcripts. By constructing UniTransModels, we analyzed AS at the whole-transcriptome level. AS events in flavonoid synthesis-related genes were identified digitally and then verified with PCR, which suggested that AS was likely to post-transcriptionally regulate the biosynthesis of flavonoids in *S. baicalensis*. Therefore, this work provides a solid foundation of genetic information to support a comprehensive understanding of *S. baicalensis* genetics and subsequent investigation of this plant at the molecular level.

## 2. Results and Discussion

### 2.1. Sequencing and Annotation

**Figure 1:**
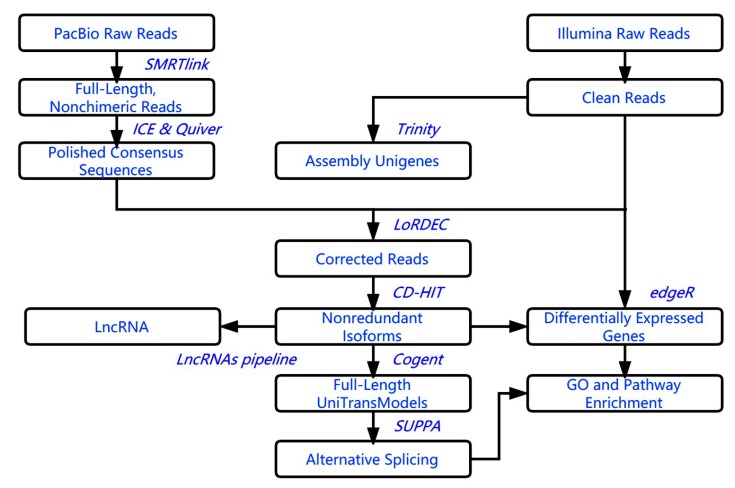
Pipeline used for analysis of hybrid sequencing data.

The PacBio Sequel platform was employed to sequence the *S. baicalensis* transcriptome. After removal of the linker and low-quality regions, a total of 532,240 reads (11.5 G data, 362,553 reads of inserts (ROIs)) with a mean read length of insert of 2.8 kb and a mean number of passes of 10 were obtained from five single-molecule real-time (SMRT) cells (Figure S1a). To generate a data set of full-length transcript models from the PacBio sequencing reads for *S. baicalensis*, we developed a computational pipeline that combined publicly available tools (Figure 1). We used the software smrtlink to process PacBio sequencing data. A total of 338,136 full-length nonchimeric (FLNC) reads ranging from 306 to 6833 bp in length were obtained, accounting for 93.3% of the total reads; the average length was 2701 bp (Figure S1b). After iterative clustering for error correction (ICE) and correction, high-quality consensus transcript sequences (28,280) and low-quality consensus transcript sequences (120,566) were acquired. The clustering results were corrected via Lordec software using a total of 371,564,626 short reads obtained from Illumina sequencing. The detailed results of correction are shown in Table S1. Insertion errors were the most common error type (Figure S2). The redundant sequences were removed from the list of consensus sequences with CD-HIT, ultimately generating 75,785 nonredundant transcripts (Table 1).

To perform comprehensive functional annotation of the *S. baicalensis* transcriptome, we annotated all nonredundant transcripts with a similarity search against protein sequences from databases including the Nonredundant (Nr), Gene Ontology (GO), Clusters of Orthologous Groups of Proteins (COG), Kyoto Encyclopedia of Genes and Genomes (KEGG), Swiss-Prot (Figure S3), and RefSeq databases. According to the statistical results, RefSeq showed the highest hit rate (97.71%), followed by Swiss-Prot (83.99%). A total of 97.97% of the transcripts were annotated with significant hits in these databases (E-value ≤ 1 × 10^−5^). However, the hit rate of Illumina sequencing in previous studies was only 70% [10]. The unassigned genes were predicted to be novel genes unique to *S. baicalensis*. The lncRNAs play crucial roles in diverse biological activities, such as the dosage compensation effect, imprinting regulation, cell cycle regulation, cell differentiation regulation, and retrotransposon silencing [24,25]. Furthermore, some studies have shown that plant lncRNAs are involved in plant responses to stress, which suggests possible involvement in plant secondary metabolism [26]. We used the lncRNA pipeline to predict the coding capability of the transcriptome and acquired 11,135 lncRNAs with a size range of 301–4296 nt and an average size of 1557 nt (Figure S4a). These lncRNAs require further investigation.

### 2.2. Comparison of the Illumina and PacBio Sequencing Results

Table S2 shows the number of reads from each replicate in Illumina RNA-seq. All short reads (371,564,626) from Illumina sequencing were assembled to yield 106,549 unigenes (N50 = 1744). PacBio sequencing yielded 75,785 nonredundant transcripts (N50 = 2794; Table 1). Homologous comparison of the assembled Illumina sequences and the PacBio sequencing transcripts showed that 33.16% (35,330) of the assembled Illumina sequences were unmapped, 48.74% (51,928) matched with an identity rate <99%, and only 18.11% (19,291) matched with an identity rate ≥99% (Figure S5). In addition, 43,155 unique transcriptional sequences of PacBio sequencing were not found in Illumina sequencing. These results indicate that the assembly of Illumina sequencing short reads may lead to errors, causing in a large proportion of transcripts in Illumina sequencing to be unable to map to PacBio sequencing data.

**Figure 2:**
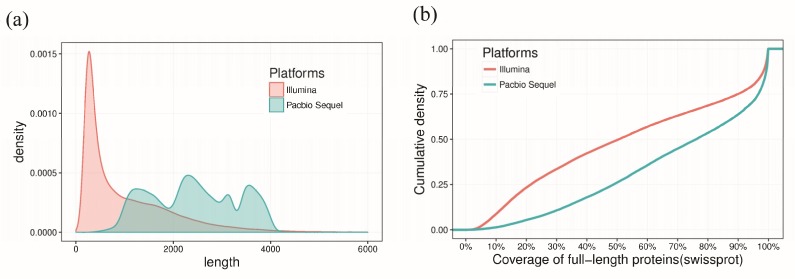
Mapping statistics for corrected long reads from PacBio sequencing and de novo assembled contigs from Illumina sequencing. (**a**) Length distribution of Iso-Seq consensus transcripts and de novo–assembled contigs from Illumina sequencing. (**b**) Cumulative density plot showing the coverage of full-length proteins (Swiss-Prot) for transcripts identified by different sequencing platforms.

In a previous study, de novo assembly of *S. baicalensis* transcripts based on Illumina HiSeq 2000 sequencing revealed fragmented contigs and showed that only approximately 23,813 (48.1%) of the total unigenes had lengths greater than 1 kb [10]. In this study, hybrid sequencing showed that 28,623 (97.27%) of the total transcripts had lengths exceeding 1 kb, suggesting the significant advantage of combining the long reads from the PacBio platform and the high-quality reads from the Illumina sequencing platform. With regard to density, the lengths of the Illumina sequencing-derived transcripts were mostly below 2 kb, much shorter than those derived from PacBio sequencing (Figure 2a). With regard to complete open reading frame (ORF) coverage, we found that compared with Illumina de novo assembled transcripts, a significantly higher percentage of PacBio sequencing consensus isoforms contained full-length ORFs (covered 100% of a full-length protein) or nearly full-length ORFs (covered >80% of a full-length protein) (Figure 2b). Overall, our results showed that compared with Illumina sequencing, PacBio sequencing yielded longer transcript sequences, more complete protein coverage, a higher annotation rate, and greater representation of gene content. Consistent with previous studies [15,16,17,26], our results also indicated that PacBio sequencing technology is an effective method for high-quality full-length transcriptomic sequencing and is suitable for follow-up analysis of genetic structures.

### 2.3. Screening and Analysis of Differentially Expressed Genes (DEGs)

**Figure 3:**
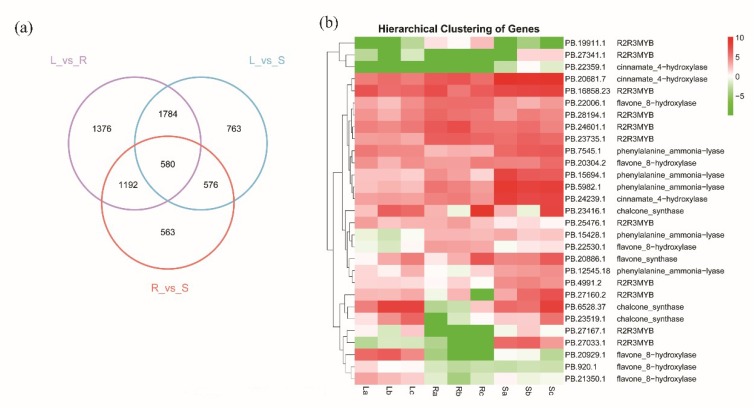
Analysis of differentially expressed genes (**a**) Venn diagrams of differentially expressed genes (DEGs) among three parts of *Scutellaria baicalensis*. (**b**) Heatmap of key genes involved in flavonoid biosynthesis in *S. baicalensis*. Note: Each square along the longitudinal axis is the value of log2 fragments per kilobase of transcript per million mapped reads (FPKM) of the corresponding gene. Red corresponds to high expression, and green corresponds to low expression.

To study differences in gene expression in different tissues, we analyzed the gene expression levels in the root, stem, and leaf of *S. baicalensis*. Fragments per kilobase of transcript per million mapped reads (FPKM) boxplots, correlation heatmap and principal component analysis (PCA) plots were created to characterize the raw and normalized data (Figures S6–S8). The results showed that there were 4932 differentially expressed genes (DEGs) between the leaf and root, 3412 of which were up-regulated in the leaf; 3703 DEGs between the root and stem, 2177 of which were up-regulated in the root; and 2911 DEGs between the leaf and stem, 2234 of which were up-regulated in the stem (Figure 3a and Table 2). As shown in Figure S9, the KEGG enrichment analysis results indicated that the DEGs highly expressed in the leaf compared with the stem and root were most enriched in the terms “Carbon metabolism”, “Starch and sucrose metabolism”, and “Photosynthesis”. The highly expressed DEGs in the root compared with the leaf and stem were mainly enriched in the terms “Plant hormone signal transduction”, “Phenylpropanoid biosynthesis”, “Flavone and flavonol biosynthesis”, etc. Studies have shown that although various parts of *S. baicalensis* produce baicalin, the highest amounts are in the roots [27]. Flavonoids are downstream metabolites of phenylpropanoid biosynthesis and are most abundant in roots and less abundant in stems and leaves. Correspondingly, we found that genes related to “Phenylpropanoid biosynthesis” and “Flavone and flavonol biosynthesis” were highly expressed in roots. As shown in Figure 3b, we also found that key flavonoid biosynthesis-related genes (e.g., PB22530.1) were highly expressed in the roots, suggesting that flavonoids are synthesized and accumulated in the roots of *S. baicalensis*.

### 2.4. Analysis of AS Events

**Figure 4:**
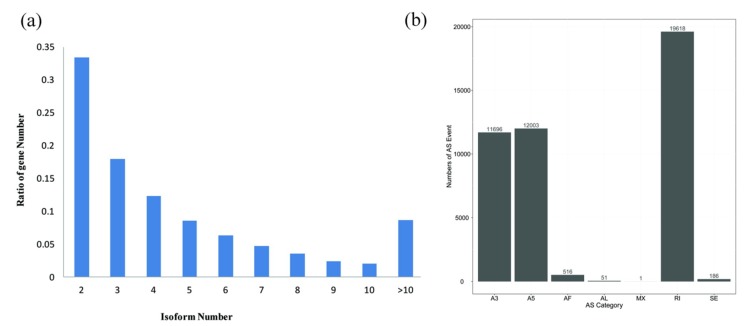
Analysis of alternative splicing (AS) events. (**a**) Distribution of isoform numbers for the genes. (**b**) Classification of AS events.

All nonredundant transcripts were assembled with the software Cogent to generate 22,948 full-length UniTransModels involving 62,562 transcripts, of which 70.6% had more than one isoform (Figure 4a). Approximately 10.7% (1729) of UniTransModels had more than 10 isoforms, and 504_0|path55 showed the highest number of isoforms (a total of 79 splicing isoforms). The total number of AS events was 44,071, including A3 (11,696), A5 (12,003), AF (516), AL (51), MX (1), IR (19,618), and SE (186) events (Figure 4b). Three dominant types of AS events (IR, A3, and A5) accounted for 98.3% of the total events, and the IR type had the highest proportion (44.5%). The mRNA involvement was as follows: A3, 11,318 transcripts; A5, 11,630 transcripts; AF, 432 transcripts; AL, 50 transcripts; MX, one transcript; IR, 18,880 transcripts; and SE, 180 transcripts. The lncRNA involvement was as follows: A3, 611 transcripts; A5, 591 transcripts; AF, 88 transcripts; AL, one transcript; MX, no transcripts; IR, 1456 transcripts; and SE, 15 transcripts. Similar to the results of the analysis above, the lncRNA isoforms were also mostly dominated by A3, A5, and IR isoforms (Figure S4b). The results showed that PacBio sequencing technology is very powerful for AS discovery.

AS analyses of *Gossypium raimondii* (40%) and *Populus trichocarpa* (45%) have also shown that the AS events are dominated by IR [28,29]. Differential intron splicing indicates synergistic up- or down-regulated IR, which is an important molecular mechanism by which *P. trichocarpa* copes with stress. IR can regulate many genes involved in cell wall metabolism, plant development, circadian rhythm, and stress responses [29]. In maize, IR can introduce termination codons to activate nonsense-mediated decay (NMD), but it can also change ORFs, leading to the production of different functional variants [30]. In addition, IR isoforms from parental genes combined with RNA may be reverse-transcribed into cDNA and then recruited into the genome to become novel genes [31]. The analysis of the *S. baicalensis* transcriptome also suggested that IR was the major type of AS, accounting for 44.5% of the total events, indicating that IR plays a vital role in the AS of *S. baicalensis*.

**Figure 5:**
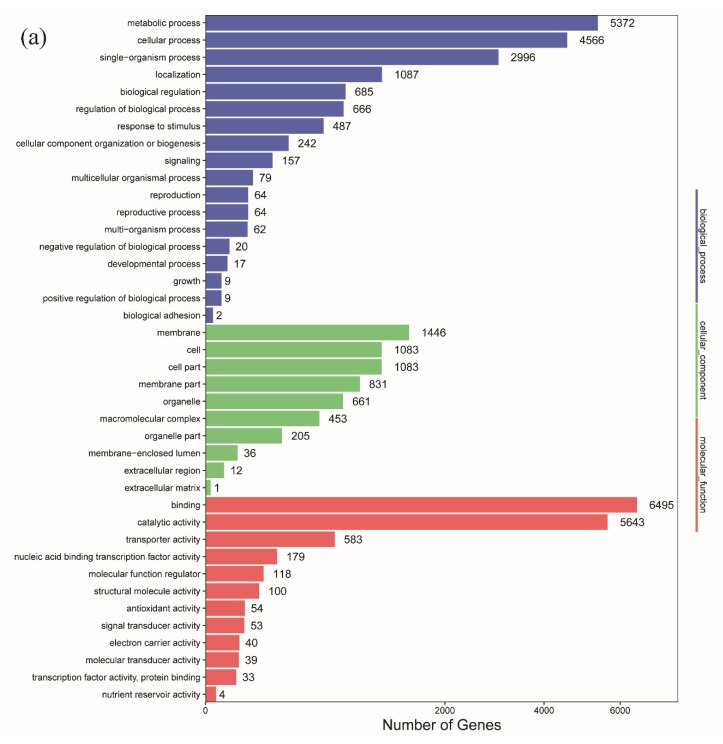

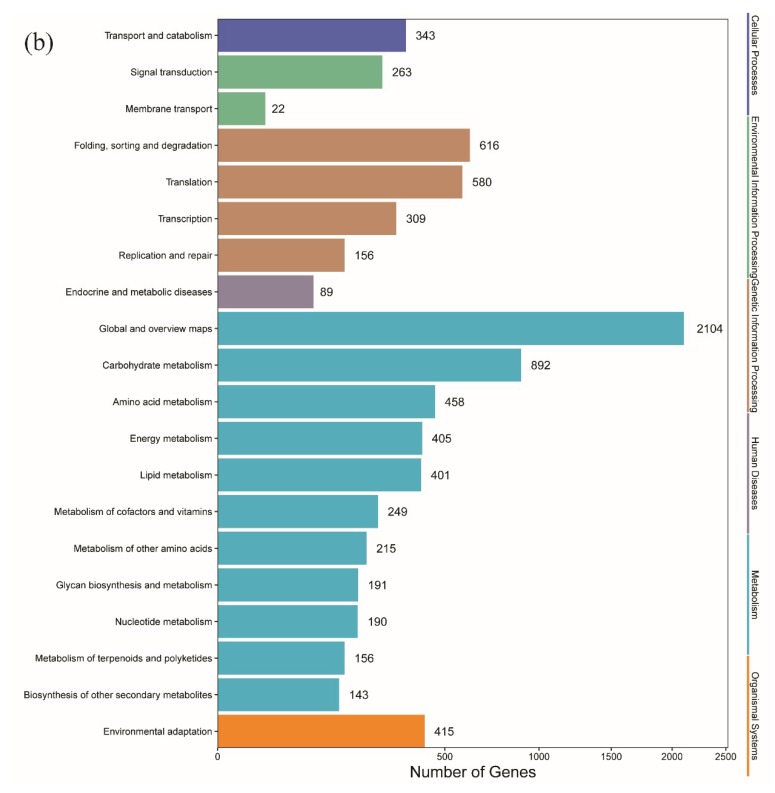
Functional annotation and classification of unigenes associated with AS events in *S. baicalensis*. (**a**) Gene Ontology (GO) enrichment; (**b**) Kyoto Encyclopedia of Genes and Genomes (KEGG) enrichment.

Previous studies have shown that AS may play important roles in secondary metabolism. For example, AS has been observed to occur in approximately 40% of the genes in the roots of *S. miltiorrhiza*, which may be related to the regulation of terpenoid synthesis [15]. One gene of *A. membranaceus* has dissimilar splicing isoforms in the leaf and root, most likely related to tissue-specific functions [16]. In two dihydroflavonol 4-reductase (*DFR*) genes, which play key roles in anthocyanin biosynthesis of the red flower variety of *Gerbera jamesonii*, splicing occurs through mutation, producing a white flower mutant [32]. GO analysis showed that 16,584 unigenes associated with AS events were assigned to 18 GO Biological Process functional terms (Figure 5a). Of these, “Metabolic process”-related unigenes were particularly enriched, and 5372 unigenes were annotated, accounting for 15.0% of the total unigenes associated with AS events. Further classification of the unigenes associated with AS events and “Metabolic process” functional terms in the GO database indicated that primary metabolism accounted for 29% of the unigenes, and four unigenes (PB.2686.2, PB.19439.1, PB.15140.1, PB.19942.1) were involved in secondary metabolism. In the Molecular Function category, large proportions of unigenes were associated with the “Binding” and “Catalytic activity” terms at 18.2% and 15.8%, respectively, indicating that different isoforms of these unigenes may play significant roles in the metabolic processes and protein-binding and enzymatic activity of *S. baicalensis*. KEGG analysis showed that 143, 20, and 103 unigenes were annotated with the “Biosynthesis of other secondary metabolites”, “Flavonoid biosynthesis”, and “Phenylpropanoid biosynthesis” terms, respectively (Figure 5b), suggesting that similar to the previous findings, AS may be involved in the metabolic processes of flavonoid synthesis-related genes.

### 2.5. Analysis of Flavonoid Biosynthesis-Related Genes

Flavonoids, the main active ingredients of *S. baicalensis*, have been extensively investigated, and some key genes in flavonoid metabolic pathways have also been cloned and characterized [33,34,35,36]. These key enzymes include phenylalanine ammonia-lyase (*PAL*), 4-coumarate: CoA ligase (*4CL*), cinnamate-4-hydroxylase (*C4H*), chalcone synthase (*CHS*), chalcone isomerase (*CHI*), flavanone-3-hydroxylase (*F3H*), flavanone-6-hydroxylase (*F6H*), flavanone-8-hydroxylase (*F8H*), and flavone synthase (*FNS*) [32]. In addition, recent studies found that the R2R3-MYB transcription factor (TF) genes might be responsible for regulating the production of flavonoids in *S. baicalensis* [37,38].

**Figure 6:**
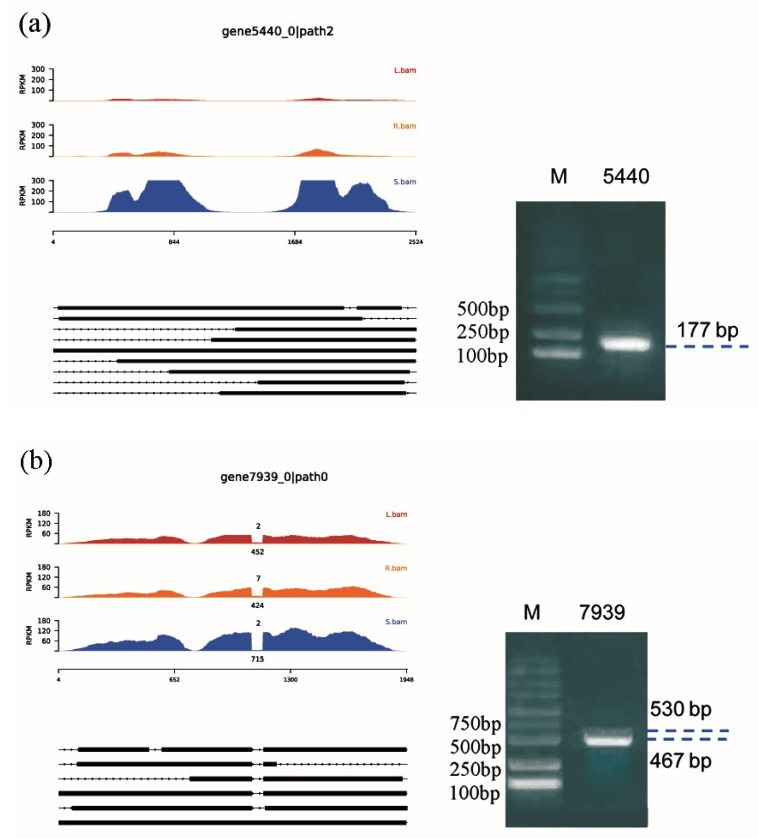

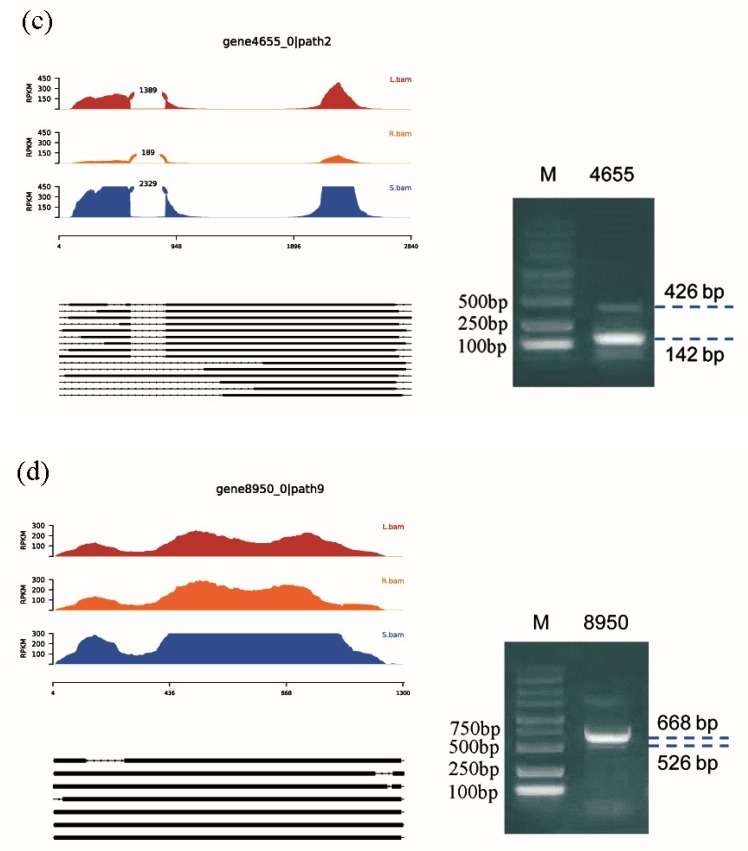
AS prediction and PCR validation of flavonoid biosynthesis-related key genes. (**a**) phenylalanine ammonia-lyase (*PAL*) gene 5440; (**b**) flavanone-8-hydroxylase (*F8H*) gene 7939; (**c**) *PAL* gene 4655; (**d**) R2R3-MYB gene 8950. Note: The alignments and coverages of Illumina reads and UniTransModels in the leaf, root, and stem are indicated in red, orange, and blue, respectively (the vertical axis represents the normalized number of supporting reads, i.e., the expression level). Below are the positional relationships between the isoform sequences obtained through PacBio sequencing and their corresponding UniTransModels (the black part indicates that at that position, the isoform was mapped to the UniTransModel, while the dashed line indicates that the isoform was not mapped to the UniTransModel). The right part displays the PCR verification results of the AS of key genes.

Through analysis of the annotated sequencing data, 188 transcript sequences of the eight key genes in flavonoid biosynthesis (except *CHI* and *F6H*) were obtained. After further analysis using Cogent, we obtained 155 transcripts corresponding to the 77 key gene-related UniTransModels. Excluding the *FNS*-coding gene, which corresponded to only one transcript, the other key genes all had more than one isoform. Finally, we performed multiple sequence alignments with protein sequences translated from distinct isoforms and analyzed the conserved domains, and we found that 94 AS events were present in five (*4CL*-, *F3H*-, *F8H*-, *PAL*-, and R2R3-MYB-coding genes) of the eight key genes involved in flavonoid synthesis. These genes produced 75 transcripts (Table S3) through AS events, including A3 (7), A5 (12), and IR (75) events. Some transcripts were obtained through more than one AS event. With regard to key genes, such as the *PAL*-encoding genes 5440 and 4665, the *F8H*-encoding gene 7939, and the R2R3-MYB-TFs-encoding gene 8950 (Figure 6), the expression patterns of the same genes in different tissues of *S. baicalensis* were essentially identical, but the expression levels differed significantly. In addition, many genes exhibited multiple isoforms. Gene 5440 and gene 7939 had nine and six isoforms, respectively. Further PCR amplification conducted with specific primers (Table S4), designed based on the flanking sequences of splicing sites using cDNA as the template (Figure 6), generated one amplicon for gene 5440; thus, the AS event was not detected via PCR assay, possibly suggesting differences in sensitivity between PCR and sequencing. The PCR assay results for other genes were consistent with those predicted by AS, and multiple amplicons consistent with the predicted sizes derived from AS were generated, which verified the authenticity of the AS events.

Multiple alignments of the protein sequences of isoforms corresponding to key genes related to flavonoid biosynthesis are shown in Figure S10. In our study, AS events were determined to be present in five key genes involved in flavonoid synthesis in both CDSs and UTRs. Below, some cases of AS events are described. Gene 7939 can encode a functional *F8H* with 510 amino acid residues. In its isoform 20304.2, an IR event occurs between nucleotides 1083 and 1145 bp of the UniTransModel and introduces a premature termination codon (PTC), probably resulting in a truncated protein. In addition, the isoforms 20665.3 and 20304.3, with A5 types of AS, also produce C-terminal truncated proteins. Gene 8950 encodes transcription factor R2R3-MYB. In its isoform 16858.14, AS event leads to a deletion of 143 nucleotides and produces a PTC at a position very close to the 5’ end of the ORF, suggesting this isoform will probably be degraded by nonsense-mediated mRNA decay pathway [39]. In the isoforms 16858.4, 16858.10, and 16858.22, AS events lead to truncated proteins compared to reference sequences, the real functions of these truncated proteins need further study. An A5-type AS event occurs in the UTR of isoform 16858.17 of the R2R3-MYB TFs, which will not affect the predicted protein sequence. However, previous studies have shown that AS in the UTR may still affect mRNA stability, localization, and expression of key genes [40]. These results suggest that AS could play important roles in flavonoid synthesis by regulating key enzymes and/or related TFs at a posttranscriptional level.

## 3. Materials and Methods

### 3.1. Materials and RNA Extraction

Three typical *S. baicalensis* plants (three years old) were chosen from the *S. baicalensis* cultivation base in Qingdao, Shandong Province, China. The roots, leaves, and stems of each plant were separated, cleaned, wrapped individually in aluminum foil, frozen, and stored in liquid nitrogen. A total of 100 g of young tissue was obtained from the root, stem, and leaf parts of each plant for high-quality DNA extraction using the CTAB method. From the RNA samples of the roots, stems and leaves extracted from the three plants, one representative sample with high RNA quality was selected for each tissue, and equal quantities of these samples were pooled. The pooled sample was used for PacBio sequencing. For Illumina sequencing, the samples were collected in triplicate from each tissue from three individuals, and nine separate cDNA libraries were constructed and sequenced for the different tissues.

### 3.2. cDNA Library Construction and Sequencing

The RNA integrity number (RIN) of the extracted RNA was determined using an Agilent 2100 bioanalyzer (Agilent, Palo Alto, CA, USA). The qualified RNA (RIN ≥ 8) was reverse-transcribed into cDNA using a SMARTer® PCR cDNA Synthesis Kit (Takara Bio USA, Inc., Mountain View, CA, USA). PCR amplification was performed using a KAPA HiFi PCR Kit (Kapa Biosystems, Wilmington, MA, USA). PCR optimization was performed to determine the optimal number of PCR cycles. For size selection, PCR amplification was performed using the optimized number of cycles, and the amplified products were fragment-sorted over a gradient of 0.5–6 kb. The sorted fragments were subjected to large-scale PCR amplification to obtain sufficient total amounts of DNA, from which a SMRTbell library was constructed using a SMRTbell Template Prep Kit 1.0 (Pacific Biosciences, Menlo Park, CA, USA). End repair was routinely conducted, and the ends of DNA fragments were linked with a stem-loop sequencing linker. The fragments that failed to link to the linker were removed using exonuclease. Nine cDNA libraries of *S. baicalensis* were obtained from the root, stem or leaf, and the 1–2 kb, 2–3 kb, and >3 kb libraries were constructed on the PacBio Sequel sequencing platform (Pacific Biosciences, Menlo Park, CA, USA). The libraries of the three fragment sizes were mixed in equal amounts and sequenced. After removal of the linker and low-quality regions, the total reads acquired in the five cells were counted. The Illumina sequencing data were generated in one lane on the HiSeq X Ten PE150 platform (Illumina, San Diego, CA, USA). Trinity software (V2.0.6) was used to assemble the Illumina sequencing data, and the minimum contig length was set to 150 bp. Then, clustering was performed using Trinity TGICL software, and sequences less than 200 bp were filtered out. All short reads (371,564,626) from Illumina sequencing were assembled and merged, and the redundant sequences were removed to obtain the final unigenes (106,549).

### 3.3. Iso-Seq Data Processing and Contig Mapping Through Two Generations of Sequencing

After the sequencing was completed, the raw data were analyzed using SMRTlink4.0. After removing the linker and low-quality regions, the postfilter polymerase reads were acquired from the raw data. The consensus sequences were clustered and generated through the ICE algorithm module of the ICE package and corrected using Quiver. The subreads from the sequences of the same polymerase reads were used to generate the ROIs, which included the consensus sequences of the 5’ primer, 3’ primer and poly-A tail and were called the full-length reads. All sequences were subjected to redundancy removal to yield nonredundant transcripts using CD-HIT v4.6 (parameters: -c 0.99, -T 6, -G 0, -aL 0.90, -AL 100, -aS 0.99, -AS 30). Both high-quality (accuracy > 0.99) and low-quality (accuracy < 0.99) consensus transcript sequences were acquired after redundancy removal. The clustering results were corrected with Lordec software using all the short reads (371,564,626) obtained from Illumina sequencing. After the correction was completed, the numbers of mismatches, insertions, and deletions were counted to calculate the average quality value. BLAST software (V2.3.0) was used to align the Illumina assembly sequences to the corrected PacBio sequences. The threshold was set to an E-value < 1 × 10^−10^, the proportions of the Illumina assembly sequences with different mapping levels to the total data were counted separately.

### 3.4. Full-Length UniTransModel Reconstruction and AS Analysis

The nonredundant transcripts were further assembled using Cogent v1.4 (https://github.com/Magdoll/Cogent), and each transcript family was reconstructed into one or several UniTransModels using a De Bruijn graph method. The nonredundant transcripts were then mapped to the UniTransModels using GMAP software. The GMAP mapping results were exported to SUPPA software (using the default parameters) to detect the AS events. The Illumina sequencing data were mapped to the UniTransModels using HISAT2 software. Based on the mapping results from GMAP and HISAT2, a Sashimi map was generated using the sashimi_plot plugin. Multiple sequence alignments were performed using the program Clustal Omega, and the conserved domains were predicted using the NCBI conserved domain prediction tool.

### 3.5. Analysis of DEGs

We used PacBio sequencing to obtain unigenes as a reference gene set, and then the Illumina sequencing reads were aligned to the PacBio sequencing unigenes using the specific sequence alignment software Bowtie 2. Using RSEM (v1.1.12) [41], the read count value of each Illumina sequencing gene was directly obtained and then transformed into the fragments per kilobase of transcript per million mapped reads (FPKM) value. Then, the DEGs between different tissue samples (roots, leaves, and stems) were detected with the standardization method TMM of the R package edgeR [42]. We performed multiple hypothesis test correction for the *p* values of the difference tests and determined the ranges of the *p* values by controlling the false discovery rate (FDR) [43]. In the analysis, a *p* value ≤ 0.05 and an FDR ≤ 0.01 were used as the thresholds for DEG screening, and an FPKM value of 0.1 was used as the threshold for judging whether a gene was expressed. An FPKM value of 1 indicated that only one RNA molecule was present in the cell, an FPKM value between 0.1 and 3.75 indicated a low gene expression level, an FPKM value between 3.75 and 15 indicated a midrange gene expression level, and an FPKM value above 15 indicated a high gene expression level. FPKM plots were used to measure the divergence between different samples from the perspective of the overall dispersion of gene expression. The correlation heatmap shows correlations of all samples. PCA plots were created to characterize the raw and normalized data.

### 3.6. Functional Annotation

Based on the 75,785 nonredundant transcripts obtained by three generations of sequencing, all DEGs were mapped to nucleic acid and protein sequence databases using the BLAST program (E-value < 1 × 10^−5^) to determine the best annotation. The protein databases included the Swiss-Prot, GO, KEGG, COG, and GenBank Nr databases; the NCBI reference sequence database (RefSeq) of high-throughput sequencing data was also used. All the annotation information was collated, and the target genes were screened out. The unigenes were finally annotated by a BLAST search against six databases, namely, the Nr database with an E-value threshold of 1 × 10^−5^, the GO database with an E-value threshold of 1 × 10^−6^, the COG database with an E-value threshold of 1 × 10^−3^, the KEGG database with an E-value threshold of 1 × 10^−10^, the Swiss-Prot database with an E-value threshold of 1 × 10^−5^, and the RefSeq database with an E-value threshold of 1 × 10^−5^.

### 3.7. Protein and lncRNA Identification

Protein predictions were performed using ORF finder. The minimum ORF length was set to 300 bp. The lncRNAs were predicted based on the acquired nonredundant transcripts using redundancy removal with the lncRNA pipeline procedure in the core program of PLEK.

### 3.8. Accession Number

All clean sequence read data were deposited in the NCBI SRA database (accession number: PRJNA515574).

## 4. Conclusions

In this study, we investigated the full-length transcriptome of *S. baicalensis* through hybrid sequencing technology. A total of 338,136 full-length nonchimeric (FLNC) reads were obtained, accounting for 93.3% of the total reads. After redundancy removal and correction with Illumina short reads, 75,785 nonredundant transcripts were generated. Using full-length or near-full-length transcripts without splicing for subsequent analyses can ensure sequence accuracy, improving the reliability of analyses such as annotation, expression, and AS analyses. Approximately 98% of the nonredundant transcripts were annotated as mRNAs encoding proteins, and 11,135 transcripts were classified as lncRNAs. DEG analysis showed that most genes related to flavonoid biosynthesis were highly expressed in the roots of *S. baicalensis*, suggesting that the *S. baicalensis* flavonoids were mainly synthesized in the roots, which was consistent with previous studies. In addition, a total of 44,071 AS events were detected, with IR accounting for the highest proportion of events at 44.5%. Ninety-four AS events were observed in five key genes related to flavonoid biosynthesis. The authenticity of some AS events was confirmed by PCR. The resulting isoforms exhibited differences in their UTRs or CDSs, indicating that AS possibly regulated flavonoid biosynthesis at the posttranscriptional level in *S. baicalensis.* This study provided not only new insights into the regulation of AS in the biosynthesis of flavonoids but also valuable genetic resources for further exploring its functional genomics in *S. baicalensis*.

## Figures and Tables

**Table 1:** *Scutellariabaicalensis* transcriptome.

Sequence	Total Number	Mean Length	N50	GC Content (%)
Non redundant_isoforms	75,785	2426	2794	41.87
lncRNA	11,135	1557	1590	43.06
mRNA	64,650	2575	2893	41.74
UniTransModel	22,948	2870	3435	41.13

**Table 2:** Number of differentially expressed genes.

Pairs	Up	Down	Total
L^1^-vs-R^2^	1520	3412	4932
R-vs-S^3^	1526	2177	3703
L-vs-S	2234	677	2911

Note: 1: leaf, 2: root, 3: stem.

## Supplementary Materials

The following are available online at https://www.mdpi.com/1422-0067/20/18/4426/s1.
