# Supplementary material for: Hybrid Sequencing of Full-Length cDNA Transcripts of the Medicinal Plant Scutellaria baicalensis
Source: Int J Mol Sci. 2019 Sep 9;20(18):4426. doi: 10.3390/ijms20184426 (PMC6770217; doi:10.3390/ijms20184426)
Supplement: Supplementary file 1 [file ijms-20-04426-s001.pdf]

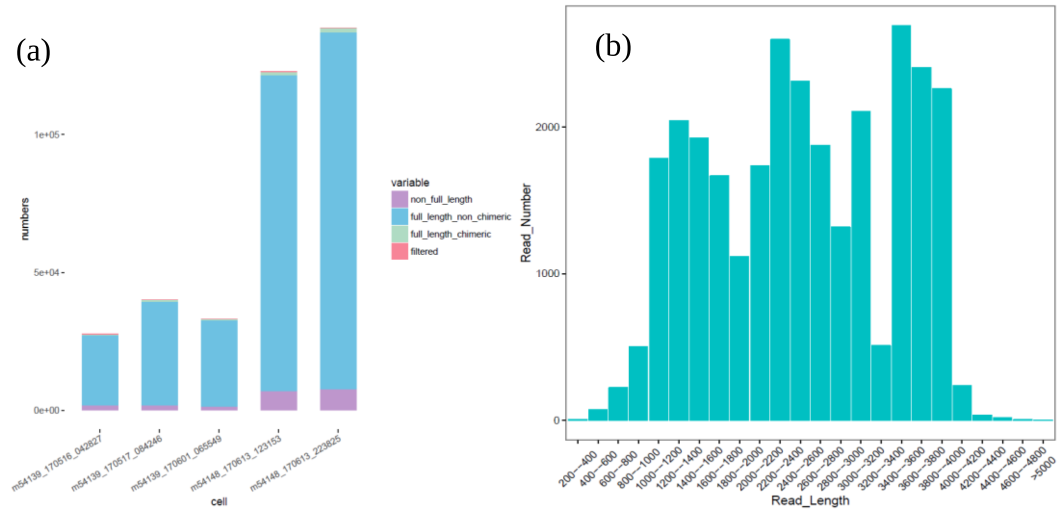


Figure S1 (a) Number of each type of reads (b) Length distribution of full-length non-chimeric reads

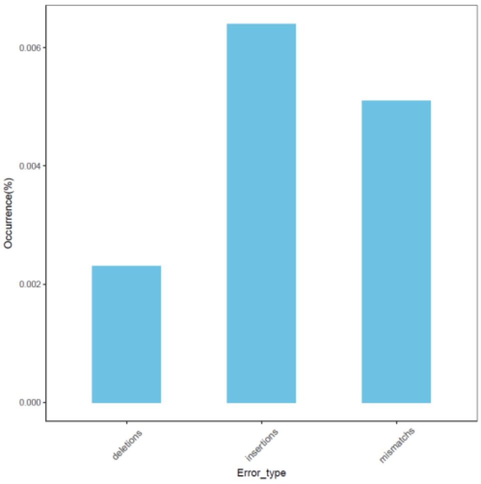


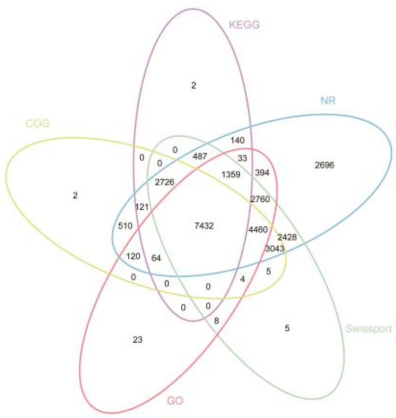


Figure S2 Error type distribution.

Figure S3 Venn diagrams of transcripts annotations.

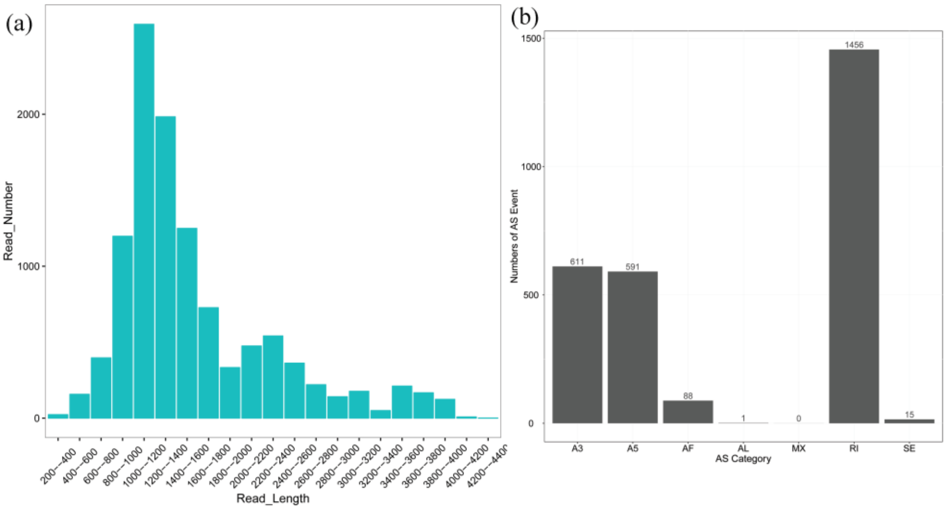


Figure S4 Analysis of lncRNAs (a) Length distribution of lncRNAs (b) Distribution of types of isoforms of lncRNAs

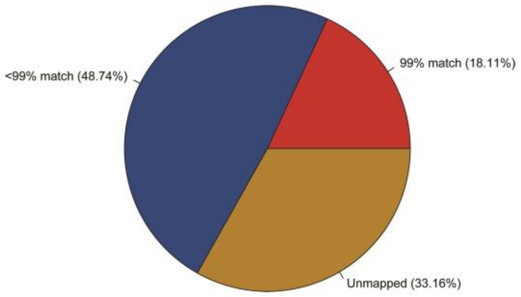


Figure S5 Pie chart of mapped vs. unmapped contigs assembled from Illumina sequencing with a 99% mapping level threshold. The unmapped value represents the proportion of Illumina sequencing contigs not aligned to PacBio corrected long reads.

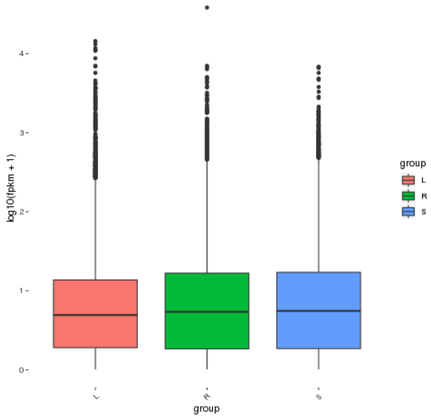


FigureS6 FPKM box plots

Note: X-axis: L, R, S group; Y-axis: log10 (FPKM+1) was used to measure the expression of the genes in different tissue samples. The box plots of each region correspond to five statistic values. The top-down five statistic values are the maximum, the top quartile, the middle quartile, the bottom quartile and the minimum, respectively.

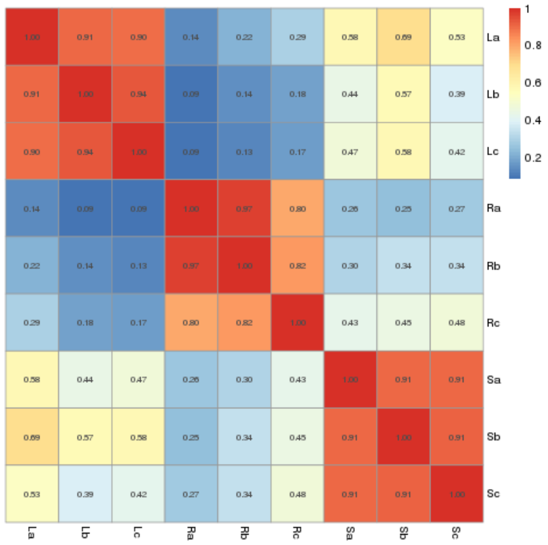


Figure S7 Correlation heatmap of all samples

Note: The gradient from blue to red indicates the correlation between the two samples from low to high

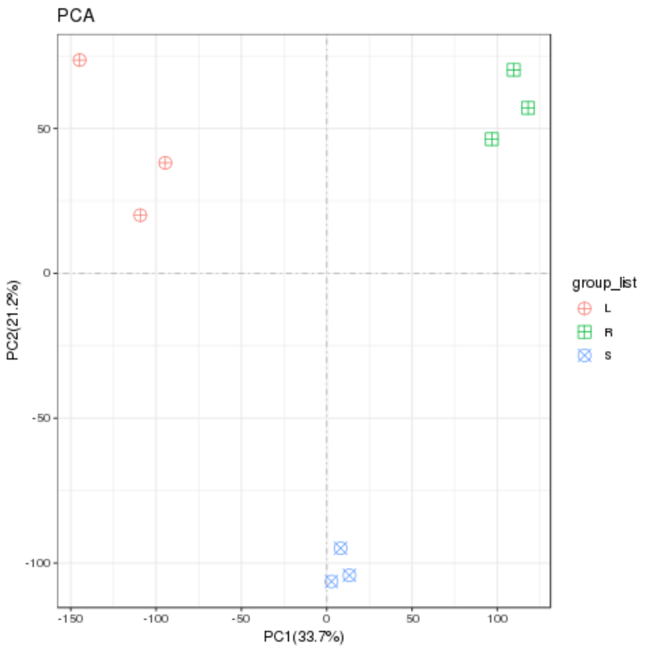


Figure S8 Principal Component Analysis of all samples

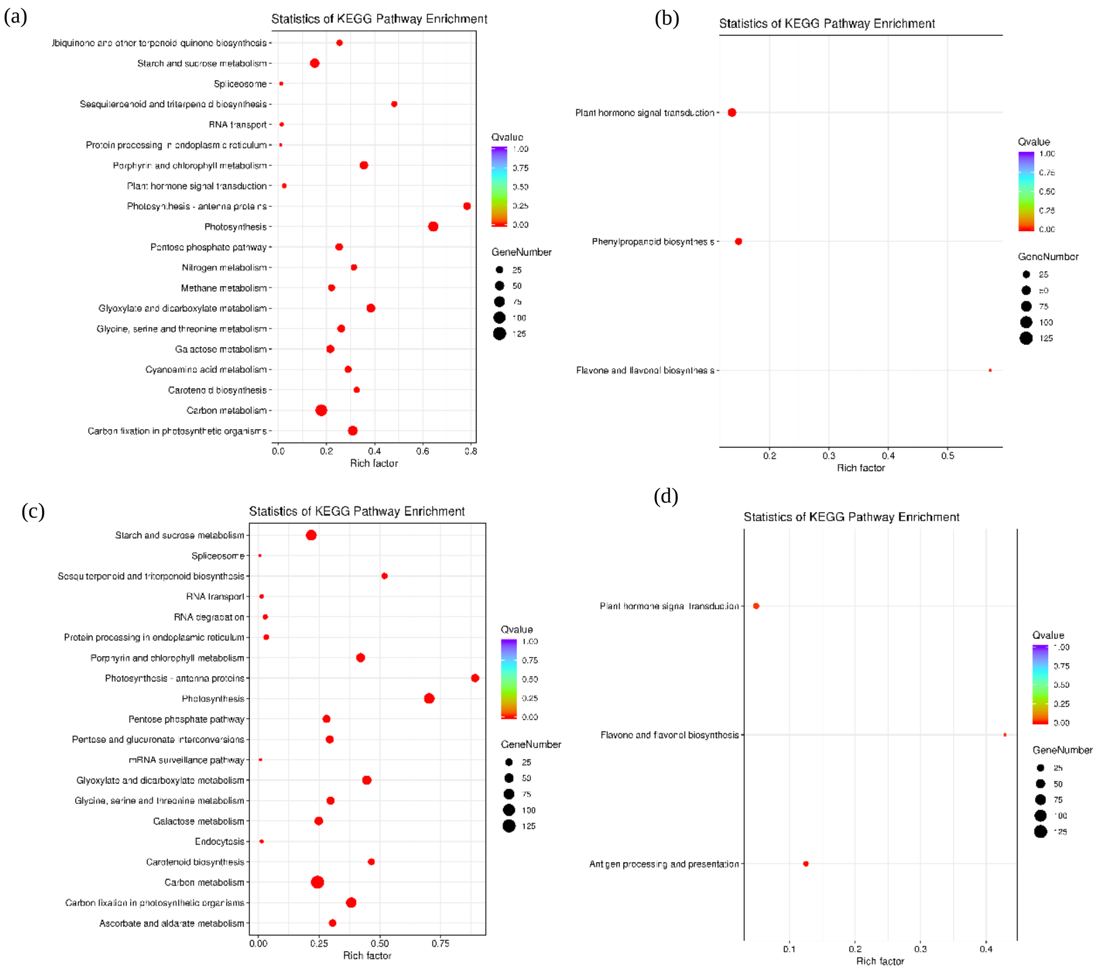


Figure S9 KEGG enrichment analysis of DEGs among different tissues of *Scutellaria baicalensis*. (a) Leaf vs. stem down (b) Leaf vs. root up (c) Leaf vs. root down (d) Root vs. stem down

| | | |
|---|---|---|
| 14211.2 | MENANSLCDDPLNWKMAAESLSGSHLDEVKRMVAEYRKAVVRLGGETLTIGQVAAVATRE | 60 |
| 5982.6 | MENANSLCDDPLNWKMAAESLSGSHLDEVKRMVAEYRKAVVRLGGETLTIGQVAAVATRE | 60 |
| 5440 | MENANSLCDDPLNWKMAAESLSGSHLNEVKRMVAEYRKAVVRLGGETLTIGQVAAVATRE | 60 |
| 5982.14 | MENANSLCDDPLNWKMAAESLSGSHLDEVKRMVAEYRRAVVRLGGETLTIGQVAAVATRE | 60 |
| 5982.8 | ------------------------------------------------------------ | 0 |
| 5982.9 | ------------------------------------------------------------ | 0 |
| 5982.10 | ------------------------------------------------------------ | 0 |
| 5982.2 | ------------------------------------------------------------ | 0 |
| 5982.3 | ------------------------------------------------------------ | 0 |
| 5982.5 | ------------------------------------------------------------ | 0 |
| 5982.4 | ------------------------------------------------------------ | 0 |

| | | |
|---|---|---|
| 14211.2 | AAVKVELAESAREGVKASSDWVMESMDKGTDSYGVTTGFGATSHRRTKQGGALQKELIRF | 120 |
| 5982.6 | AAVKVELAESAREGVKASSDWVMESMDKGTDSYGVTTGFGATSHRRTKQGGALQKELIRF | 120 |
| 5440 | AAVKVELAESAREGVKASSDWVMESMDKGTDSYGVTTGFGATSHRRTKQGGALQKELIRF | 120 |
| 5982.14 | AAVKVELAESAREGVKASSDWVMESMDKGTDSYGVTTGFGATSHRRTKQGGALQKELIRF | 120 |
| 5982.8 | ------------------------------------------------------------ | 0 |
| 5982.9 | ------------------------------------------------------------ | 0 |
| 5982.10 | ------------------------------------------------------------ | 0 |
| 5982.2 | ------------------------------------------------------------ | 0 |
| 5982.3 | ------------------------------------------------------------ | 0 |
| 5982.5 | ------------------------------------------------------------ | 0 |
| 5982.4 | ------------------------------------------------------------ | 0 |

| | | |
|---|---|---|
| 14211.2 | LNAGIFGKGSDADHTLPHTATRASMLVRINTLLQGYSGIRFEVLEAITKFLNHNITPCLP | 180 |
| 5982.6 | LNAGIFGKGSDADHTLPHTATRASMLVRINTLLQGYSGIRFEVLEAITKFLNHNITPCLP | 180 |
| 5440 | LNAGIFGKDSDADHTLPHTATRASMFVRINTLLQGYSGIRFEVLEAITKFLNHNITPCLP | 180 |
| 5982.14 | LNAGIFGKGSDADHTLPHTATRASMLVRINTLLQGYSGIRFEVLEAITKFLNHNITPCLP | 180 |
| 5982.8 | ------------------------MLVRINTLLQGYSGIRFEVLEAITKFLNHNITPCLP | 36 |
| 5982.9 | ------------------------------------------------------------ | 0 |
| 5982.10 | ------------------------------------------------------------ | 0 |
| 5982.2 | ------------------------------------------------------------ | 0 |
| 5982.3 | ------------------------------------------------------------ | 0 |
| 5982.5 | ------------------------------------------------------------ | 0 |
| 5982.4 | ------------------------------------------------------------ | 0 |

| | | |
|---|---|---|
| 14211.2 | LRGTITASGDLVPLSYIAGLLLGRPNSKATGPNGEALGPAQAFALAGIDS---GFFELQP | 237 |
| 5982.6 | LRGTITASGDLVPLFYIAGLLLGRPNSKATGPNGEALGPAQAFALAGIDS---GFFELQP | 237 |
| 5440 | LRGTITASGDLVPLSYIAGLLLGRPNSKATGPNGEALGPAQAFSLAGIDSGFSGFFELQP | 240 |
| 5982.14 | LRGTITASGDLVPLSYIAGLLLGRPNSKATGPNGEALGPAQAFALAGIDS---GFFELQP | 237 |
| 5982.8 | LRGTITASGDLVPLSYIAGLLLGRPNSKATGPNGEALGPAQAFALAGIDS---GFFELQP | 93 |
| 5982.9 | ------------------------------------------------------------ | 0 |
| 5982.10 | ------------------------------------------------------------ | 0 |
| 5982.2 | ------------------------------------------------------------ | 0 |
| 5982.3 | ------------------------------------------------------------ | 0 |
| 5982.5 | ------------------------------------------------------------ | 0 |
| 5982.4 | ------------------------------------------------------------ | 0 |

| | | |
|---|---|---|
| 14211.2 | KEGLALVNGTAVGSGPASMVLFEANILAVLSEVMSAVFAEVMQGKPEFTDHLTHKLKHHP | 297 |
| 5982.6 | KEGLALVNGTAVGSGLASMVLFEANILAVLSEVMSAVFAEVMQGKPEFTDHLTHKLKHHP | 297 |
| 5440 | KEGLALVNGTAVGSGLASMVLFEANILAVLSEVMSAVFAEVMQGKPEFTDHLTHKLKHHP | 300 |
| 5982.14 | KEGLALVNGTAVGSGPASMVLFEANILAVLSEVMSAVFAEVMQGKPEFTDHLTHKLKHHP | 297 |
| 5982.8 | KEGLALVNGTAVGSGPASMVLFEANILAVLSEVMSAVFAEVMQGKPEFTDHLTHKLKHHP | 153 |
| 5982.9 | ------------------MVLFEANILAVLSEVMSAVFAEVMQGKPEFTDHLTHKLKHHP | 42 |
| 5982.10 | ------------------MVLFEANILAVLSEVMSAVFAEVMQGKPEFTDHLTHKLKHHP | 42 |
| 5982.2 | ------------------------------------------------------------ | 0 |
| 5982.3 | ------------------------------------------------------------ | 0 |
| 5982.5 | ------------------------------------------------------------ | 0 |
| 5982.4 | ------------------------------------------------------------ | 0 |

| | | |
|---|---|---|
| 14211.2 | GQIEAAAIMEHILDGSSIVKEAEKVHELDPLQKPKQDRYALRTSPQWLGPQIEVIRAATK | 357 |
| 5982.6 | GQIEAAAIMEHILDGSSIVKEAEKVHELDPLQKPKQDRYALRTSPQWLGPQIEVIRAATK | 357 |
| 5440 | GQIEAAAIMEHILDGSSIVKEAEKVHELDPLQKPKQDRYALRTSPQWLGPQIEVIRAATK | 360 |

```
5982.14         GQIEAAAIMEHILDGSSIVKEAEKVHELDPLQKPKQDRYALRTSPQWLGPQIEVIRAATK    357
5982.8          GQIEAAAIMEHILDGSSIVKEAEKVHELDPLQKPKQDRYALRTSPQWLGPQIEVIRAATK    213
5982.9          GQIEAAAIMEHILDGSSIVKEAEKVHELDPLQKPKQDRYALRTSPQWLGPQIEVIRAATK    102
5982.10         GQIEAAAIMEHILDGSSIVKEAEKVHELDPLQKPKQDRYALRTSPQWLGPQIEVIRAATK    102
5982.2          ------------------------------------------------------------    0
5982.3          ------------------------------------------------------------    0
5982.5          ------------------------------------------------------------    0
5982.4          ------------------------------------------------------------    0

14211.2         SIEREINSVNDNPLIDVSRNKALHGGNFQGTPIGVSMDNTRLAIASIGKLMFAQFSELVN    417
5982.6          SIEREINSVNDNPLIDVSRNKALHGGNFQGTPIGVSMDNTRLAIASIGKLMFAQFSELVN    417
5440            SIEREINSVNDNPLIDVSRNKALHGGNFQGTPIGVSMDNTRLAIASIGKLMFAQFSELVN    420
5982.14         SIEREINSVNDNPLIDVSRNKALHGGNFQGTPIGVSMDNTRLAIASIGKLMFAQFSELVN    417
5982.8          SIEREINSVNDNPLIDVSRNKALHGGNFQGTPIGVSMDNTRLAIASIGKLMFAQFSELVN    273
5982.9          SIEREINSVNDNPLIDVSRNKALHGGNFQGTPIGVSMDNTRLAIASIGKLMFAQFSELVN    162
5982.10         SIEREINSVNDNPLIDVSRNKALHGGNFQGTPIGVSMDNTRLAIASIGKLMFAQFSELVN    162
5982.2          ------------------------------------MDNTRLAIASIGKLMFAQFSELVN    24
5982.3          ------------------------------------MDNTRLAIASIGKLMFAQFSELVN    24
5982.5          ------------------------------------MDNTRLAIASIGKLMFAQFSELVN    24
5982.4          ------------------------------------------------------------    0


14211.2         DFYNNGLPSNLSGGRNPSLDYGFKGAEIAMAAYCSELQFLANPVTNHVQSAEQHNQDVNS    477
5982.6          DFYNNGLPSNLSGGRNPSLDYGFKGAEIAMAAYCSELQFLANPVTNHVQSAEQHNQDVNS    477
5440            DFYNNGLPSNLSGGRNPSLDYGFKGAEIAMAAYCSELQFLANPVTNHVQSAEQHNQDVNS    480
5982.14         DFYNNGLPSNLSGGRNPSLDYGFKGAEIAMAAYCSELQFLANPVTNHVQSAEQHNQDVNS    477
5982.8          DFYNNGLPSNLSGGRNPSLDYGFKGAEIAMAAYCSELQFLANPVTNHVQSAEQHNQDVNS    333
5982.9          DFYNNGLPSNLSGGRNPSLDYGFKGAEIAMAAYCSELQFLANPVTNHVQSAEQHNQDVNS    222
5982.10         DFYNNGLPSNLSGGRNPSLDYGFKGAEIAMAAYCSELQFLANPVTNHVQSAEQHNQDVNS    222
5982.2          DFYNNGLPSNLSGGRNPSLDYGFKGAEIAMAAYCSELQFLANPVTNHVQSAEQHNQDVNS    84
5982.3          DFYNNGLPSNLSGGRNPSLDYGFKGAEIAMAAYCSELQFLANPVTNHVQSAEQHNQDVNS    84
5982.5          DFYNNGLPSNLSGGRNPSLDYGFKGAEIAMAAYCSELQFLANPVTNHVQSAEQHNQDVNS    84
5982.4          -----------------------------MAAYCSELQFLANPVTNHVQSAEQHNQDVNS    31
                                             *******************************

14211.2         LGLISSRKTGEAVEILKLMSSTFLVGLCQAIDLRHLEENLKASVKNTVSLVAKKVLTMGQ    537
5982.6          LGLISSRKTGEAVEILKLMSSTFLVGLCQAIDLRHLEENLKASVKNTVSLVAKKVLTMGQ    537
5440            LGLISSRKTGEAVEILKLMSSTFLVGLCQAIDLRHLEENLKALVKNTVSLVAKKVLTMGQ    540
5982.14         LGLISSRKTGEAVEILKLMSSTFLVGLCQAIDLRHLEENLKASVKNTVSLVAKKVLTMGQ    537
5982.8          LGLISSRKTGEAVEILKLMSSTFLVGLCQAIDLRHLEENLKASVKNTVSLVAKKVLTMGQ    393
5982.9          LGLISSRKTGEAVEILKLMSSTFLVGLCQAIDLRHLEENLKASVKNTVSLVAKKVLTMGQ    282
5982.10         LGLISSRKTGEAVEILKLMSSTFLVGLCQAIDLRHLEENLKASVKNTVSLVAKKVLTMGQ    282
5982.2          LGLISSRKTGEAVEILKLMSSTFLVGLCQAIDLRHLEENLKASVKNTVSLVAKKVLTMGQ    144
5982.3          LGLISSRKTGEAVEILKLMSSTFLVGLCQAIDLRHLEENLKASVKNTVSLVAKKVLTMGQ    144
5982.5          LGLISSRKTGEAVEILKLMSSTFLVGLCQAIDLRHLEENLKASVKNTVSLVAKKVLTMGQ    144
5982.4          LGLISSRKTGEAVEILKLMSSTFLVGLCQAIDLRHLEENLKASVKNTVSLVAKKVLTMGQ    91
                ****************************************** *****************

14211.2         NGELHPSRFCEKDLLKVVDREHVFGYIDDPCSVNYPLMLKLRQVLVDHALANSVHDEDEK    597
5982.6          NGELHPSRFCEKDLLKVVDREHVFGYIDDPCSVNYPLMLKLRQVLVDHALANSVHDEDEK    597
5440            NGELHPSRFCEKDLLKVVDREHVFGYIDDPCSVNYPLMLKLRQVLVDHALANSVHDEDEK    600
5982.14         NGELHPSRFCEKDLLKVVDREHVFGYIDDPCSVNYPLMLKLRQVLVDHALANSVHDEDEK    597
5982.8          NGELHPSRFCEKDLLKVVDREHVFGYIDDPCSVNYPLMLKLRQVLVDHALANSVHDEDEK    453
5982.9          NGELHPSRFCEKDLLKVVDREHVFGYIDDPCSVNYPLMLKLRQVLVDHALANSVHDEDEK    342
5982.10         NGELHPSRFCEKDLLKVVDREHVFGYIDDPCSVNYPLMLKLRQVLVDHALANSVHDEDEK    342
5982.2          NGELHPSRFCEKDLLKVVDREHVFGYIDDPCSVNYPLMLKLRQVLVDHALANSVHDEDEK    204
5982.3          NGELHPSRFCEKDLLKVVDREHVFGYIDDPCSVNYPLMLKLRQVLVDHALANSVHDEDEK    204
5982.5          NGELHPSRFCEKDLLKVVDREHVFGYIDDPCSVNYPLMLKLRQVLVDHALANSVHDEDEK    204
5982.4          NGELHPSRFCEKDLLKVVDREHVFGYIDDPCSVNYPLMLKLRQVLVDHALANSVHDEDEK    151
                ************************************************************

14211.2         NFGSSIFHKIGAFEEELKALLPSL------------------------------------    621
5982.6          NFGSSIFHKIGAFEEELKALLPKEVESARLEVETGKAAIGNRIKNCRSLPLYKFVREEAG    657
5440            NFGSSIFHKIGAFEEELKALLPKEVESARLEVETGKAAIGNRIKNCRSLPLYKFVREEAG    660
5982.14         NFGSSIFHKIGAFEEELKALLPKEVESARLEVETGKAAIGNRIKNCRSLPLYKFVREEAG    657
5982.8          NFGSSIFHKIGAFEEELKALLPKEVESARLEVETGKAAIGNRIKNCRSLPLYKFVREEAG    513
5982.9          NFGSSIFHKIGAFEEELKALLPKEVESARLEVETGKAAIGNRIKNCRSLPLYKFVREEAG    402
```

```
5982.10      NFGSSIFHKIGAFEEELKALLPKEVESARLEVETGKAAIGNRIKNCRSLPLYKFVREEAG     402
5982.2       NFGSSIFHKIGAFEEELKALLPKEVESARLEVETGKAAIGNRIKNCRSLPLYKFVREEAG     264
5982.3       NFGSSIFHKIGAFEEELKALLPKEVESARLEVETGKAAIGNRIKNCRSLPLYKFVREEAG     264
5982.5       NFGSSIFHKIGAFEEELKALLPKEVESARLEVETGKAAIGNRIKNCRSLPLYKFVREEAG     264
5982.4       NFGSSIFHKIGAFEEELKALLPKEVESARLEVETGKAAIGNRIKNCRSLPLYKFVREEAG     211
             *********************.

14211.2      ----------------------------------------------     621
5982.6       TGFLT-----------------------------------------     662
5440         TGFLTGEKDRSPGEEFDKVFTAICEGKLIDPFLECLKEWNGAPIPIC     707
5982.14      TGFLTGEKDRSPGEEFDKVFTAICEGKLIDPLLECLKEWNGAPIPIC     704
5982.8       TGFLTGEKDRSPGEEFDKVFTAICEGKLIDPLLECLKEWNGAPIPIC     560
5982.9       TGFLTGEKDRSPGEEFDKVFTAICEGKLIDPLLECLKEWNGAPIPIC     449
5982.10      TGFLTGEKDRSPGEEFDKVFTAICEGKLIDPLLECLKEWNGAPIPIC     449
5982.2       TGFLTGEKDRSPGEEFDKVFTAICEGKLIDPLLECLKEWNGAPIPIC     311
5982.3       TGFLTGEKDRSPGEEFDKVFTAICEGKLIDPLLECLKEWNGAPIPIC     311
5982.5       TGFLTGEKDRSPGEEFDKVFTAICEGKLIDPLLECLKEWNGAPIPIC     311
5982.4       TGFLTGEKDRSPGEEFDKVFTAICEGKLIDPLLECLKEWNGAPIPIC     258


16858.4      MAARNESDRIKGPWSPEEDELLQRLVEKHGPRNWSLISKSISGRSGKSCRLRWCNQLSPQ     60
16858.22     MAARNESDRIKGPWSPEEDELLQRLVEKHGPRNWSLISKSIPGRSGKSCRLRWCNQLSPQ     60
16858.10     MAARNESDRIKGPWSPEEDDLLQRLVEKHGPRNWSLISKSIPGRSGKSCRLRWCNQLSPQ     60
8950         MAARNESDRIKGPWSPEEDELLQRLVEKHGPRNWSLISKSISGRSGKSCRLRWCNQLSPQ     60
16858.14     ------------------------------------------------------------     0
16858.17     MAARNESDRIKGPWSPEEDELLQRLVEKHGPRNWSLISKSIPGRSGKSCRLRWCNQLSPQ     60
16858.20     MAARNESDRIKGPWSPEEDELLQRLVEKHGPRNWSLISKSIPGRSGKSCRLRWCNQLSPQ     60


16858.4      VEHPAFTPEEDETIIRAHAKFGNKWATIARLLSGRTDNAIKNHWNSTLKRKCVSMSEEFN     120
16858.22     VEHRAFTPEEDETIIRAHAKFGNKWATIARLLSGRTDNAIKNHWNSTLKRKCVSMSEEFN     120
16858.10     VEHRAFTPEEDETIIRAHAKFGNKWATIARLLSGRTDNAIKNHWNSTLKRKCVSMSEEFN     120
8950         VEHPAFTPEEDETIIRAHAKFGNKWATIARLLSGRTDNAIKNHWNSTLKRKCVSMSEEFN     120
16858.14     ------------------------------------------------------MSEEFN     6
16858.17     VEHRAFTPEEDETIIRAHAKFGNKWATIARLLSGRTDNAIKNHWNSTLKRKCVSMSEEFN     120
16858.20     VEHRAFTPEEDETIIRAHAKFGNKWATIARLLSGRTDNAIKNHWNSTLKRKCVSMSEEFN     120
                                                                   ******

16858.4      NFDPDAQPPLKRSASVGPGSNISTSGFCFNPGRPTESDVSDSSHSGHHVYRPIPRTGGIS     180
16858.22     NFDPDAQPPLKRSASVGPGSNISTSGFCFNPGSPTESDVSDSSHSGHHVYRPIPRTGGIS     180
16858.10     NFDPRRSAAAQAVR----------------------------------------------     134
8950         NFDPDAQPPLKRSASVGPGSNISTSGFCFNPGRPTESDVSDSSHSGHHVYRPIPRTGGIS     180
16858.14     NFDPDARPPLKRSASVGPGSNISTSGFCFNPGSPTESDVSDSSHSGHHVYRPIPRTGGIS     66
16858.17     NFDPDAQPPLKRSASVGPGSNISTSGFCFNPGSPTESDVSDSSHSGHHVYRPIPRTGGIS     180
16858.20     NFDPDAQPPLKRSASVGPGSNISTSGFCFNPGSPTESDVSDSSHSGHHVYRPIPRTGGIS     180
             ****       :

16858.4      PPDHQPDPITSLSLSLPGCDTNPTEPSNPDPITVLDSALIPGPVLPIYPIQMIPSHPPPP     240
16858.22     PPDHQPDPITSLSLSLPGCDTNPTEPSNPDPITVLDSALIPGPVLPIYPIQMIPSHPPPP     240
16858.10     ------------------------------------------------------------     134
8950         PPDHQPDPITSLSLSLPGCDTNPTEPSNPDPITVLDSALIPGPVLPIYPIQMIPSHPPPP     240
16858.14     PPDHQPDPITSLSLSLPGCDTNPTEPSNPDPITVLDSALIPGPVLPIYPIQMIPSHPPPP     126
16858.17     PPDHQPDPITSLSLSLPGCDTNPTEPSNPDPITVLDSGLIPGPVLPIYPIQMIPSHPPPP     240
16858.20     PPDHQPDPITSLSLSLPGCDTNPTEPSNPDPITVLDSALIPGPVLPIYPIQMIPSHPPPP     240


16858.4      -PPPAPTTGRYGHGGGETVLQH--------------------------------------     261
16858.22     PPPPAPTAGRYGHGGGETVLQH--------------------------------------     262
16858.10     ------------------------------------------------------------     134
8950         PPPPPQQPADMGTAAEKQFFSTEFLSVMQEMVKKEVRNYMAGIEQNGLCMQSEAIRNAVV     300
16858.14     PPPPPQQPADMGTAAEKQFFSTEFLSVMQEMVKKEVRNYMAGIEQNGLCMQSEAIRNAVV     186
16858.17     PPPPPQQPADMGTAAEKQFFSTEFLSVMQEMVKKEVRNYMAGIEQNGLCMQSEAIRNAVV     300
16858.20     PPPPPQQPADMGTAAEKQFFSTEFLSVMQEMVKKEVRNYMAGIEQNGLCMQSEAIRNAVV     300


16858.4      ---------    261
16858.22     ---------    262
```

```
16858.10      ---------      134
8950          KRMGISKID      309
16858.14      KRMGISKI-      194
16858.17      KRMGISKID      309
16858.20      KRMGISKID      309

12545.15      MAPAVEVSHRSNGFCVQLSDPLNWGAAAEALKGSHLDEVKRMVEEFRNPVVKIGGENLTI      60
12545.16      ------------------------------------------------------------      0
4655          MAPAVEVSHRSNGFCVQLSDPLNWGAAAEALKGSHLDEVKRMVEEFRNPVVKIGGENLTI      60
12545.17      MAPAVEVSHRSNGFCVQLSDPLNWGAAAEALKGSHLDEVKRMVEEFRNPVVKIGGENLTI      60
12545.4       ------------------------------------------------------------      0
12545.5       MAPAVEVSHRSNGFCVQLSDPLNWGAAAEALKGSHLDEVKRMVEEFRNPVVKIGGENLTI      60
12545.6       MAPAVEVSHRSNGFCVQLSDPLNWGAAAEALKGSHLDEVKRMVEEFRNPVVKIGGENLTI      60
14470.1       MAPAVEVSHRSNGFCVQLSDPLNWGAAAEALKGSHLDEVKRMVEEFRNPVVKIGGENLTI      60
12545.12      -----------------------------------------MVEEFRNPVVKIGGENLTI      19
12545.11      ------------------------------------------------------------      0
12545.18      ------------------------------------------------------------      0
12545.9       ------------------------------------------------------------      0
12545.10      ------------------------------------------------------------      0
12545.7       ------------------------------------------------------------      0
12545.1       ------------------------------------------------------------      0
12545.8       ------------------------------------------------------------      0


12545.15      AQVAAIASRDNAVAVELAESARAGVKASSDWVMDSMNKGTDSYGVTTGFGATSHRRTKQG      120
12545.16      ------------------------------------------------------------      0
4655          AQVAAIASRDNAVAVELAESARAGVKASSDWVMDSMNKGTDSYGVTTGFGATSHRRTKQG      120
12545.17      AQVAAIASRDNAVAVELAESARAGVKASSDWVMDSMNKGTDSYGVTTGFGATSHRRTKQG      120
12545.4       --------------------------------MDSMNKGTDSYGVTTGFGATSHRRTKQG      28
12545.5       AQVAAIASRDNAVAVELAESARAGVKASSDWVMDSMNKGTDSYGVTTGFGATSHRRTKQG      120
12545.6       AQVAAIASRDNAVAVELAESARAGVKASSDWVMDSMNKGTDSYGVTTGFGATSHRRTKQG      120
14470.1       AQVAAIASRDNAVAVELAESARAGVKASSDWVMDSMNKGTDSYGVTTGFGATSHRRTKQG      120
12545.12      AQVAAIASRDNAVAVELAESARAGVKASSDWVMDSMNKGTDSYGVTTGFGATSHRRTKQG      79
12545.11      ------------------------------------------------------------      0
12545.18      ------------------------------------------------------------      0
12545.9       ------------------------------------------------------------      0
12545.10      ------------------------------------------------------------      0
12545.7       ------------------------------------------------------------      0
12545.1       ------------------------------------------------------------      0
12545.8       ------------------------------------------------------------      0


12545.15      GALQKELIRFLNAGIFGKGTEGCHTLPHTATRAAMLVRINTLLQGYSGIRFEILEVLTKF      180
12545.16      ----------------------------------MLVRINTLLQGYSGIRFEILEALTKF      26
4655          GALQKELIRFLNAGIFEKGTEGCHTLPHTATRAAMLVRIITLLQGYSGIRFEILEALTKF      180
12545.17      GALQKELIRFLNAGIFEKGTEGCHTLPHTATRAAMLVRIITLLQGYSGIRFEILEALTKF      180
12545.4       GALQKELIRFLNAGIFGKGTEGCHTLPHTATRAAMLVRINTLLQGYSGIRFEILEALTKF      88
12545.5       GALQKELIRFLNAGIFGKGTEGCHTLPHTATRAAMLVRINTLLQGYSGIRFEILEALTKF      180
12545.6       GALQKELIRFLNAGIFGKGTEGCHTLPHTATRAAMLVRINTLLQGYSGIRFEILEALTKF      180
14470.1       GALQKELIRFLNAGIFGKGTEGCHTLPHTATRAAMLVRINTLLQGYSGIRFEILEALTKF      180
12545.12      GALQKELIRFLNAGIFGKGTEGCHTLPHTATRAAMLVRINTLLQGYSGIRFEILEALTKF      139
12545.11      ----------------------------------MLVRINTLLQGYSGIRFEILEALTKF      26
12545.18      ----------------------------------MLVRINTLLQGYSGIRFEILEALTKF      26
12545.9       ------------------------------------------------------------      0
12545.10      ------------------------------------------------------------      0
12545.7       ------------------------------------------------------------      0
12545.1       ------------------------------------------------------------      0
12545.8       ------------------------------------------------------------      0


12545.15      LNHNITPCLPLRGTITASGDLVPLSYIAGLLTGRPNSKAVGPKGEELNAEEAFKLAGVSG      240
12545.16      LNHNITPCLPLRGTITASGDLVPLSYIAGLLTGRPNSKAVGPKGEELNAEEAFKLAGVSG      86
4655          LKHNITPCLPLRGTITASGDLVPLSYIAGLLTGRPNSKAVGPKGEELNAEEAFKLAGVSG      240
12545.17      LKHNITPCLPLRGTITASGDLVPLSYIAGLLTGRPNSKAVGPKGEELNAEEAFKLAGVSG      240
12545.4       LNHNITPCLPLRGTITASGDLVPLSYIAGLLTGRPNSKAVGPKGEELNAEEAFKLAGVSG      148
12545.5       LNHNITPCLPLRGTITASGDLVPLSYIAGLLTGRPNSKAVGPKGEELNAEEAFKLAGVSG      240
12545.6       LNHNITPCLPLRGTITASGDLVPLSYIAGLLTGRPNSKAVGPKGEELNAEEAFKLAGVSG      240
```

```
14470.1     LNHNITPCLPLRGTITASGDLVPLSYIAGLLTGRPNSKAVGPKGEELNAEEAFKLAGVSG     240
12545.12    LNHNITPCLPLRGTITASGDLVPLSYIAGLLTGRPNSKAVGPKGEELNAEEAFKLAGVSG     199
12545.11    LNHNITPCLPLRGTITASGDLVPLSYIAGLLTGRPNSKAVGPKGEELNAEEAFKLAGVSG     86
12545.18    LNHNITPCLPLRGTITASGDLVPLSYIAGLLTGRPNSKAVGPKGEELNAEEAFKLAGVSG     86
12545.9     ------------------------------------------------------------     0
12545.10    ------------------------------------------------------------     0
12545.7     ------------------------------------------------------------     0
12545.1     ------------------------------------------------------------     0
12545.8     ------------------------------------------------------------     0


12545.15    GFFELQPKEGLALVNGTAVGSGLASIALYEANVLALLAEVMSAIFAEVMNGKPEFTDHLT     300
12545.16    GFFELQPKEGLALVNGTAVGSGLASIALYEANVLALLAEVMSAIFAEVMNGKPEFTDHLT     146
4655        GFFELQPKEGLALVNGTAVGSGLASIALYEANVLALLAEVMSAIFAEVMNGKPEFTDHLT     300
12545.17    GFFELQPKEGLALVNGTAVGSGLASIALYEANVLALLAEVMSAIFAEVMNGKPEFTDHLT     300
12545.4     GFFELQPKEGLALVNGTAVGSGLASIALYEANVLALLAEVMSAIFAEVMNGKPEFTDHLT     208
12545.5     GFFELQPKEGLALVNGTAVGSGLASIALYEANVLALLAEVMSAIFAEVMNGKPEFTDHLT     300
12545.6     GFFELQPKEGLALVNGTAVGSGLASIALYEANVLALLAEVMSAIFAEVMNGKPEFTDHLT     300
14470.1     GFFELQPKEGLALVNGTAVGSGLASIALYEANVLALLAEVMSAIFAEVMNGKPEFTDHLT     300
12545.12    GFFELQPKEGLALVNGTAVGSGLASIALYEANVLALLAEVMSAIFAEVMNGKPEFTDHLT     259
12545.11    GFFELQPKEGLALVNGTAVGSGLASIALYEANVLALLAEVMSAIFAEVMNGKPEFTDHLT     146
12545.18    GFFELQPKEGLALVNGTAVGSGLASIALYEANVLALLAEVMSAIFAEVMNGKPEFTDHLT     146
12545.9     ----------------------------------------MSAIFAEVMNGKPEFTDHLT     20
12545.10    ----------------------------------------MSAIFAEVMNGKPEFTDHLT     20
12545.7     ------------------------------------------------MNGKPEFTDHLT     12
12545.1     ------------------------------------------------------------     0
12545.8     ------------------------------------------------------------     0

12545.15    HKLKHHPGQIEAAAIMEHILDGSAYVKAAQKMHEMDPLQKPKQDRYALRTSPQWLGPQIE     360
12545.16    HKLKHHPGQIEAAAIMEHILDGSAYVKAAQKMHEMDPLQKPKQDRYALRTSPQWLGPQIE     206
4655        HKLKHHPGQIEAAAIMEHILDGSAYVKAAQKMHEMDPLQKPKQDRYALRTSPQWLGPQIE     360
12545.17    HKLKHHPGQIEAAAIMEHILDGSAYVKAAQKMHEMDPLQKPKQDRYALRTSPQWLGPQIE     360
12545.4     HKLKHHPGQIEAAAIMEHILDGSAYVKAAQKMHEMDPLQKPKQDRYALRTSPQWLGPQIE     268
12545.5     HKLKHHPGQIEAAAIMEHILDGSAYVKAAQKMHEMDPLQKPKQDRYALRTSPQWLGPQIE     360
12545.6     HKLKHHPGQIEAAAIMEHILDGSAYVKAAQKMHEMDPLQKPKQDRYALRTSPQWLGPQIE     360
14470.1     HKLKHHPGQIEAAAIMEHILDGSAYVKAAQKMHEMDPLQKPKQDRYALRTSPQWLGPQIE     360
12545.12    HKLKHHPGQIEAAAIMEHILDGSAYVKAAQKMHEMDPLQKPKQDRYALRTSPQWLGPQIE     319
12545.11    HKLKHHPGQIEAAAIMEHILDGSAYVKAAQKMHEMDPLQKPKQDRYALRTSPQWLGPQIE     206
12545.18    HKLKHHPGQIEAAAIMEHILDGSAYVKAAQKMHEMDPLQKPKQDRYALRTSPQWLGPQIE     206
12545.9     HKLKHHPGQIEAAAIMEHILDGSAYVKAAQKMHEMDPLQKPKQDRYALRTSPQWLGPQIE     80
12545.10    HKLKHHPGQIEAAAIMEHILDGSAYVKAAQKMHEMDPLQKPKQDRYALRTSPQWLGPQIE     80
12545.7     HKLKHHPGQIEAAAIMEHILDGSAYVKAAQKMHEMDPLQKPKQDRYALRTSPQWLGPQIE     72
12545.1     ------------------------------------------------------------     0
12545.8     ------------------------------------------------------------     0

12545.15    VIRTATKMIEREINSVNDNPLIDVSRNKAIHGGNFQGTPIGVSMDNTRLAIAAIGKLMFA     420
12545.16    VIRTATKMIEREINSVNDNPLIDVSRNKAIHGGNFQGTPIGVSMYNTRLAIAAIGKLMFA     266
4655        VIRTATKMIEREIHSVNDNPLIDVSRNKAIHGGNFQGTPIGVSMDNTRLAIAAIGKLMFA     420
12545.17    VIRTATKMIEREIHSVNDNPLIDVSRNKAIHGGNFQGTPIGVSMDNTRLAIAAIGKLMFA     420
12545.4     VIRTATKMIEREINSVNDNPLIDVSRNKAIHGGNFQGTPIGVSMDNTRLAIAAIGKLMFA     328
12545.5     VIRTATKMIEREINSVNDNPLIDVSRNKAIHGGNFQGTPIGVSMDNTRLAIAAIGKLMFA     420
12545.6     VIRTATKMIEREINSVNDNPLIDVSRNKAIHGGNFQGTPIGVSMDNTRLAIAAIGKLMFA     420
14470.1     VIRTATKMIEREINSVNDNPLIDVSRNKAIHGGNFQGTPIGVSMDNTRLAIAAIGKLMFA     420
12545.12    VIRTATKMIEREINSVNDNPLIDVSRNKAIHGGNFQGTPIGVSMDNTRLAIAAIGKLMFA     379
12545.11    VIRTATKMIEREINSVNDNPLIDVSRNKAIHGGNFQGTPIGVSMDNTRLAIAAIGKLMFA     266
12545.18    VIRTATKMIEREINSVNDNPLIDVSRNKAIHGGNFQGTPIGVSMDNTRLAIAAIGKLMFA     266
12545.9     VIRTATKMIEREINSVNDNPLIDVSRNKAIHGGNFQGTPIGVSMDNTRLAIAAIGKLMFA     140
12545.10    VIRTATKMIEREINSVNDNPLIDVSRNKAIHGGNFQGTPIGVSMDNTRLAIAAIGKLMFA     140
12545.7     VIRTATKMIEREINSVNDNPLIDVSRNKAIHGGNFQGTPIGVSMDNTRLAIAAIGKLMFA     132
12545.1     -------MIEREINSVNDNPLIDVSRNKAIHGGNFQGTPIGVSMDNTRLAIAAIGKLMFA     53
12545.8     -------------------------------------------MDNTRLAIAAIGKLMFA     17
                                                       * ***************

12545.15    QFSELVNDFYNNGLPSNLSGGRNPSLDYGFKGSEIAMASYCSELQFLANPVTNHVQSAEQ     480
12545.16    QFSELVNDFYNNGLPSNLSGGRNPSLDYGFKGSEIAMASYCSELQFLANPVTNHVQSAEQ     326
4655        QFSELVNDFYNNGLPSNLSGGRNPSLDYGFKGSEIAMASYCSELQFLANPVTNHVQSAEQ     480
12545.17    QFSELVNDFYNNGLPSNLSGGRNPSLDYGFKGSEIAMASYCSELQFLANPVTNHVQSAEQ     480
```

```
12545.4     QFSELVNDFYNNGLPSNLSGGRNPSLDYGFKGSEIAMASYCSELQFLANPVTNHVQSAEQ    388
12545.5     QFSELVNDFYNNGLPSNLSGGRNPSLDYGFKGSEIAMASYCSELQFLANPVTNHVQSAEQ    480
12545.6     QFSELVNDFYNNGLPSNLSGGRNPSLDYGFKGSEIAMASYCSELQFLANPVTNHVQSAEQ    480
14470.1     QFSELVNDFYNNGLPSNLSGGRNPSLDYGFKGSEIAMASYCSELQFLANPVTNHVQSAEQ    480
12545.12    QFSELVNDFYNNGLPSNLSGGRNPSLDYGFKGSEIAMASYCSELQFLANPVTNHVQSAEQ    439
12545.11    QFSELVNDFYNNGLPSNLSGGRNPSLDYGFKGSEIAMASYCSELQFLANPVTNHVQSAEQ    326
12545.18    QFSELVNDFYNNGLPSNLSGGRNPSLDYGFKGSEIAMASYCSELQFLANPVTNHVQSAEQ    326
12545.9     QFSELVNDFYNNGLPSNLSGGRNPSLDYGFKGSEIAMASYCSELQFLANPVTNHVQSAEQ    200
12545.10    QFSELVNDFYNNGLPSNLSGGRNPSLDYGFKGSEIAMASYCSELQFLANPVTNHVQSAEQ    200
12545.7     QFSELVNDFYNNGLPSNLSGGRNPSLDYGFKGSEIAMASYCSELQFLANPVTNHVQSAEQ    192
12545.1     QFSELVNDFYNNGLPSNLSGGRNPSLDYGFKGSEIAMASYCSELQFLANPVTNHVQSAEQ    113
12545.8     QFSELVNDFYNNGLPSNLSGGRNPSLDYGFKGSEIAMASYCSELQFLANPVTNHVQSAEQ    77
            ************************************************************

12545.15    HNQDVNSLGLISSRKTVEALDILKLMSSTYLVALCQAVDLRHLEENLRLAVKNTVSQLAK    540
12545.16    HNQDVNSLGLISSRKTVAALDILKLMSSTYLVALCQAVDLRHLEENLRLAVKNTVSQLAK    386
4655        HNQDVNSLGLISSRKTVEALDILKLMSSTYLVALCQAVDLRHLEENLRLAVKNTVSQLAK    540
12545.17    HNQDVNSLGLISSRKTVEALDILKLMSSTYLVALCQAVDLRHLEENLRLAVKNTVSQLAK    540
12545.4     HNQDVNSLGLISSRKTVEALDILKLMSSTYLVALCQAVDLRHLEENLRLAVKNTVSQLAK    448
12545.5     HNQDVNSLGLISSRKTVEALDILKLMSSTYLVALCQAVDLRHLEENLRLAVKNTVSQLAK    540
12545.6     HNQDVNSLGLISSRKTVEALDILKLMSSTYLVALCQAVDLRHLEENLRLAVKNTVSQLAK    540
14470.1     HNQDVNSLGLISSRKTVEALDILKLMSSTYLVALCQAVDLRHLEENLRLAVKNTVSQLAK    540
12545.12    HNQDVNSLGLISSRKTVEALDILKLMSSTYLVALCQAVDLRHLEENLRLAVKNTVSQLAK    499
12545.11    HNQDVNSLGLISSRKTVEALDILKLMSSTYLVALCQAVDLRHLEENLRLAVKNTVSQLAK    386
12545.18    HNQDVNSLGLISSRKTVEALDILKLMSSTYLVALCQAVDLRHLEENLRLAVKNTVSQLAK    386
12545.9     HNQDVNSLGLISSRKTVEALDILKLMSSTYLVALCQAVDLRHLEENLRLAVKNTVSQLAK    260
12545.10    HNQDVNSLGLISSRKTVEALDILKLMSSTYLVALCQAVDLRHLEENLRLAVKNTVSQLAK    260
12545.7     HNQDVNSLGLISSRKTVEALDILKLMSSTYLVALCQAVDLRHLEENLRLAVKNTVSQLAK    252
12545.1     HNQDVNSLGLISSRKTVEALDILKLMSSTYLVALCQAVDLRHLEENLRLAVKNTVSQLAK    173
12545.8     HNQDVNSLGLISSRKTVEALDILKLMSSTYLVALCQAVDLRHLEENLRLAVKNTVSQLAK    137
            ***************** ******************************************

12545.15    RTLTMGANGELHPSRFCEKDLLRVVDREYVFAYIDDPCSANYPLMQKLRQVLVDHALKNG    600
12545.16    RTLTMGANGELHPSRFCEKDLLRVVDREYVFAYIDDPCSANYPLMQKLRQVLVDHALKNG    446
4655        RTLTMGANGELHPSRFCEKDLLRVVDREYVFAYIDDPCSANYPLMQKLRQVLVDHALKNG    600
12545.17    RTLTMGANGELHPSRFCEKDLLRVVDREYVFAYIDDPCSANYPLMQKLRQVLVDHALKNG    600
12545.4     RTLTMGANGELHPSRFCEKDLLRVVDREYVFAYIDDPCSANYPLMQKLRQVLVDHALKNG    508
12545.5     RTLTMGANGELHPSRFCEKDLLRVVDREYVFAYIDDPCSANYPLMQKLRQVLVDHALKNG    600
12545.6     RTLTMGANGELHPSRFCEKDLLRVVDREYVFAYIDDPCSANYPLMQKLRQVLVDHALKNG    600
14470.1     RTLTMGANGELHPSRFCEKDLLRVVDREYVFAYIDDPCSANYPLMQKLRQVLVDHALKNG    600
12545.12    RTLTMGANGELHPSRFCEKDLLRVVDREYVFAYIDDPCSANYPLMQKLRQVLVDHALKNG    559
12545.11    RTLTMGANGELHPSRFCEKDLLRVVDREYVFAYIDDPCSANYPLMQKLRQVLVDHALKNG    446
12545.18    RTLTMGANGELHPSRFCEKDLLRVVDREYVFAYIDDPCSANYPLMQKLRQVLVDHALKNG    446
12545.9     RTLTMGANGELHPSRFCEKDLLRVVDREYVFAYIDDPCSANYPLMQKLRQVLVDHALKNG    320
12545.10    RTLTMGANGELHPSRFCEKDLLRVVDREYVFAYIDDPCSANYPLMQKLRQVLVDHALKNG    320
12545.7     RTLTMGANGELHPSRFCEKDLLRVVDREYVFAYIDDPCSANYPLMQKLRQVLVDHALKNG    312
12545.1     RTLTMGANGELHPSRFCEKDLLRVVDREYVFAYIDDPCSANYPLMQKLRQVLVDHALKNG    233
12545.8     RTLTMGANGELHPSRFCEKDLLRVVDREYVFAYIDDPCSANYPLMQKLRQVLVDHALKNG    197
            ************************************************************

12545.15    ENEKNASTSIFQKIEAFEEELKALLPKEVESARMALEARNPAVPNRITECRSYPLYKFIR    660
12545.16    ENEKNASTSIFQKIEAFEEELKALLPKEVESARMALEAGNPAVPNRITECRSYPLYKFIR    506
4655        ENEKNASTSIFQKIEAFEEELKALLPKEVESARMALEAGNPAVPNRITECRSYPLYKFIR    660
12545.17    ENEKNASTSIFQKIEAFEEELKALLPKEVESARMALEAGNPAVPNRITECRSYPLYKFIR    660
12545.4     ENEKNASTSIFQKIEAFEEELKALLPKEVESARMALEAGNPAVPNRITECRSYPLYKFIR    568
12545.5     ENEKNASTSIFQKIEAFEEELKALLPKEVESARMALEAGNPAVPNRITECRSYPLYKFIR    660
12545.6     ENEKNASTSIFQKIEAFEEELKALLPKEVESARMALEAGNPAVPNRITECRSYPLYKFIR    660
14470.1     ENEKNASTSIFQKIEAFEEELKALLPKEVESARMALEAGNPAVPNRITECRSYPLYKFIR    660
12545.12    ENEKNASTSIFQKIEAFEEELKALLPKEVESARMALEAGNPAVPNRITECRSYPLYKFIR    619
12545.11    ENEKNASTSIFQKIEAFEEELKALLPKEVESARMALEAGNPAVPNRITECRSYPLYKFIR    506
12545.18    ENEKNASTSIFQKIEAFEEELKALLPKEVESARMALEAGNPAVPNRITECRSYPLYKFIR    506
12545.9     ENEKNASTSIFQKIEAFEEELKALLPKEVESARMALEAGNPAVPNRITECRSYPLYKFIR    380
12545.10    ENEKNASTSIFQKIEAFEEELKALLPKEVESARMALEAGNPAVPNRITECRSYPLYKFIR    380
12545.7     ENEKNASTSIFQKIEAFEEELKALLPKEVESARMALEAGNPAVPNRITECRSYPLYKFIR    372
12545.1     ENEKNASTSIFQKIEAFEEELKALLPKEVESARMALEAGNPAVPNRITECRSYPLYKFIR    293
12545.8     ENEKNASTSIFQKIEAFEEELKALLPKEVESARMALEAGNPAVPNRITECRSYPLYKFIR    257
            ************************************** *********************
```

```
12545.15     EEAGTEFLTGRR---------------------------------------      672
12545.16     EEAGTEFLTGEKGTSPGEECDKVFTALTNGFIIDPLLTCLEAWNGAPLPIC      557
4655         EEAGTEFLTGEKVTSPGEECDKVFTALTNGFIIDPLLTCLGAWNGAPLPIC      711
12545.17     EEAGTEFLTGEKVTSPGEECDKVFTALTNGFIIDPLLTCLGAWNGAPLPIC      711
12545.4      EEAGTEFLTGEKVTSPGEECDKVFTALTNGFIIDPLLTCLEAWNGAPLPIC      619
12545.5      EEAGTEFLTGEKVTSPGEECDKVFTALTNGFIIDPLLTCLEAWNGAPLPIC      711
12545.6      EEAGTEFLTGEKVTSPGEECDKVFTALTNGFIIDPLLTCLEAWNGAPLPIC      711
14470.1      EEAGTEFLTGEKVTSPGEECDKVFTALTNGFIIDPLLTCLEAWNGAPLPIC      711
12545.12     EEAGTEFLTGEKVTSPGEECDKVFTALTNGFIIDPLLTCLEAWNGAPLPIC      670
12545.11     EEAGTEFLTGEKVTSPGEECDKVFTALTNGFIIDPLLTCLEAWNGAPLPIC      557
12545.18     EEAGTEFLTGEKVTSPGEECDKVFTALTNGFIIDPLLTCLEAWNGAPLPIC      557
12545.9      EEAGTEFLTGEKVTSPGEECDKVFTALTNGFIIDPLLTCLEAWNGAPLPIC      431
12545.10     EEAGTEFLTGEKVTSPGEECDKVFTALTNGFIIDPLLTCLEAWNGAPLPIC      431
12545.7      EEAGTEFLTGEKVTSPGEECDKVFTALTNGFIIDPLLTCLEAWNGAPLPIC      423
12545.1      EEAGTEFLTGEKVTSPGEECDKVFTALTNGFIIDPLLTCLEAWNGAPLPIC      344
12545.8      EEAGTEFLTGEKVTSPGEECDKVFTALTNGFIIDPLLTCLEAWNGAPLPIC      308
             **********.:

20665.1     ----MKELAKRFDKIFESIIDQRLKMKVQVGTESKDFLQVLLSLKD-NGDSKTPFTMTHL      55
20304.1     VQRKMKELAKRFDKIFESIIDQRLKMKGQVGTESKDFLQVLLQLKD-NGDSKTPFTMTHL      108
20665.2     VQRKMKELAKRFDKIFESIIDQRLKMKGQVGTESKDFLQVLLQLKD-NGDSKTPFTMTHL      157
7939        VQRKMKVLAKRFDKIFESIIDQRLKMKGQVGTESKDFLQVLLQLKD-NGDSKTPFTMTHL      299
20304.3     VQRKMKVLAKRFDKIFESIIDQRLKMKGQVGTESKDFLQVLLQLKD-NGDSKTPFTMTHL      299
20304.2     VQRKMKVLAKRFDKIFESIIDQRLKMKGQVGTESKDFLQVLLQLKD-NGDSKTPFTMTHL      299
20665.3     VQRKMKELAKRFDKIFESIIDQRLKMKGQVGTESKDFLQVLLQLKD-NGDSKTPFTMTHL      299
                ** ******************** **************.*****************

20665.1     KALLMDMVVGGTDTTSNTVEFALAEIMKKPQILKKVQQELEIVVGEDKIVEESHITKLPY      115
20304.1     KALLMDMVVGGTDTTSNTVEFALAEIMKKPQILKKVQQELEIVVGKDKIVEESHITKLPY      168
20665.2     KALLMDMVVGGTDTTSNTVEFALAEIMKKPQILKKVQQELEIVVGKDKIVEESHITKLPY      217
7939        KALLMDMVVGGTDTTSNTVEFALAEIMKKPQILKKVQQELEIVVGEDKIVEESHITKLPY      359
20304.3     KALLMDMVVGGTDTTSNTVEFALAEIMKK-------------------------------      328
20304.2     KALLMVSSLSLSLS------LSLSHLND--------------------------------      321
20665.3     KALLM-------------------------------------------------------      304
            *****

20665.1     LHTVMKEVLRLHPALPLLVPHCPSMTSNVAGYTIPKGSRVFINVWAIHRDPSIWENPLEF      175
20304.1     LHTVMKEVLRLHPALPLLVPHCPSMTSNVAGYTIPKGSRVFINVWAIHRDPSIWENPLEF      228
20665.2     LHTVMKEVLRLHPALPLLVPHCPSMTSNVAGYTIPKGSRVFINVWAIHRDPSIWENPLEF      277
7939        LHTVMKEVLRLHPALPLLVPHCPSMTSNVAGYTIPKGSRVFINVWAIHRDPSIWENPLEF      419
20304.3     ------------------------------------------------------------      328
20304.2     ------------------------------------------------------------      321
20665.3     ------------------------------------------------------------      304


20665.1     NPERFSDGKWDYSGNDFSYFPFGSGRMICAGIAMGERMF7939SLASLVHSLSGVCLQARSW      235
20304.1     NPERFSDGKWDYSGNDFSYFPFGSGRRICAGIAMGERMF7939SLASLVHSFEWSLPAGEKL      288
20665.2     NPERFSDGKWDYSGNDFSYFPFGSGRRICAGIAMGERMF7939SLASLVHSFEWSLPAGEKL      337
7939        NPERFSDGKWDYSGNDFSYFPFGSGRMICAGIAMGERMF7939SLASLVHSLSWSLPAGEKL      479
20304.3     ------------------------------------------------------------      328
20304.2     ------------------------------------------------------------      321
20665.3     ------------------------------------------------------------      304


20665.1     TCLRSLGEF---------------------- 244
20304.1     DLSEKFGIVLKKNAPLVAIPTPRLSDPTLYE 319
20665.2     DLSEKFGIVLKKNAPLVAIPTPRLSDPTLYE 368
7939        DLSEKFGIVLKKNAPLVAIPTPRLSDPTLYE 510
20304.3     ------------------------------- 328
20304.2     ------------------------------- 321
20665.3     ------------------------------- 304
```

Figure S10 Multiple alignment of protein sequences of isoforms corresponded to key genes related to flavonoid biosynthesis in *Scutellaria baicalensis*

Table S1 Statistics of errors of two types of correction

| Correction Type | Ratio of mapping base/ total base（%） | Mismatch | | Deletion | | Insertion | |
|---|---|---|---|---|---|---|---|
| | | No. of bases | Ratio (%) | No. of bases | Ratio (%) | No. of bases | Ratio (%) |
| Self-corrected | 59.97 | 91,726 | 0.0415 | 10,949 | 0.0050 | 20,128 | 0.0050 |
| Illumina -corrected | 62.14 | 80,529 | 0.0351 | 5,758 | 0.0025 | 6,075 | 0.0025 |

Table S2 Statistics of number of reads from each replicate in Illumina RNA-seq

| Sample | Read Length(bp) | No. of Clean reads | No. of Clean Bases | Q30 Rate (%) |
|---|---|---|---|---|
| La | 150 | 43,330,053 | 6,300,058,391 | 86.36 |
| Lb | 150 | 48,326,188 | 6,996,365,095 | 86.26 |
| Lc | 150 | 44,637,776 | 6,561,085,654 | 90.74 |
| Ra | 150 | 42,784,651 | 6,253,629,269 | 85.81 |
| Rb | 150 | 39,274,282 | 5,720,351,097 | 86.55 |
| Rc | 150 | 34,810,082 | 5,087,411,044 | 88.43 |
| Sa | 150 | 39,873,764 | 5,783,768,897 | 87.67 |
| Sb | 150 | 39,743,026 | 5,756,046,456 | 86.68 |
| Sc | 150 | 38,784,804 | 5,631,379,767 | 89.76 |

Table S3 AS statistics of transcripts corresponded to the key genes related to flavonoid biosynthesis in *Scutellaria baicalensis*

| Transcript | Key gene | UniTransModel | AS_type |
|---|---|---|---|
| PB.11197.1 | flavones 3-hydroxylase | 4055_0丨path0 | RI |
| PB.11197.2 | flavones 3-hydroxylase | 4055_0丨path1 | RI |
| PB.11197.3 | flavone 3-hydroxylase | 4055_0丨path0 | RI |
| PB.12545.1 | phenylalanine ammonia-lyase | 4655_0丨path1 | RI |
| PB.12545.10 | phenylalanine ammonia-lyase | 4655_0丨path0 | RI |
| PB.12545.11 | phenylalanine ammonia-lyase | 4655_0丨path2 | RI |
| PB.12545.12 | phenylalanine ammonia-lyase | 4655_0丨path6 | RI,A5 |
| PB.12545.14 | phenylalanine ammonia-lyase | 4655_0丨path6 | RI |
| PB.12545.15 | phenylalanine ammonia-lyase | 4655_0丨path2 | RI,A5 |
| PB.12545.16 | phenylalanine ammonia-lyase | 4655_0丨path1 | RI,A3,A5 |
| PB.12545.17 | phenylalanine ammonia-lyase | 4655_0丨path6 | RI,A5 |
| PB.16858.19 | R2R3MYB | 8950_0丨path7 | RI |
| PB.16858.2 | R2R3MYB | 8950_0丨path11 | RI |
| PB.16858.20 | R2R3MYB | 8950_0丨path9 | RI |

<table>
<tr><td>PB.16858.21</td><td>R2R3MYB</td><td>8950_0|path11</td><td>RI</td></tr>
<tr><td>PB.16858.22</td><td>R2R3MYB</td><td>8950_0|path6</td><td>RI,A3,A5</td></tr>
<tr><td>PB.16858.23</td><td>R2R3MYB</td><td>8950_0|path11</td><td>RI</td></tr>
<tr><td>PB.16858.3</td><td>R2R3MYB</td><td>8950_0|path11</td><td>RI</td></tr>
<tr><td>PB.16858.4</td><td>R2R3MYB</td><td>8950_0|path9</td><td>RI,A3,A5</td></tr>
<tr><td>PB.16858.6</td><td>R2R3MYB</td><td>8950_0|path11</td><td>RI</td></tr>
<tr><td>PB.16858.7</td><td>R2R3MYB</td><td>8950_0|path11</td><td>RI</td></tr>
<tr><td>PB.16858.8</td><td>R2R3MYB</td><td>8950_0|path11</td><td>RI</td></tr>
<tr><td>PB.17903.2</td><td>4-CoumarateCoA ligases(4CL)</td><td>8071_0|path0</td><td>RI</td></tr>
<tr><td>PB.17903.3</td><td>4-CoumarateCoA ligases(4CL)</td><td>8071_0|path0</td><td>RI</td></tr>
<tr><td>PB.17907.1</td><td>4-CoumarateCoA ligases(4CL)</td><td>6946_0|path3</td><td>RI</td></tr>
<tr><td>PB.18647.1</td><td>4-CoumarateCoA ligases(4CL)</td><td>6946_0|path3</td><td>RI</td></tr>
<tr><td>PB.18647.3</td><td>4-CoumarateCoA ligases(4CL)</td><td>6946_0|path3</td><td>RI</td></tr>
<tr><td>PB.18647.4</td><td>4-CoumarateCoA ligases(4CL)</td><td>6946_0|path3</td><td>RI</td></tr>
<tr><td>PB.18647.5</td><td>4-CoumarateCoA ligases(4CL)</td><td>6946_0|path2</td><td>RI</td></tr>
<tr><td>PB.19775.1</td><td>4-CoumarateCoA ligases(4CL)</td><td>8071_0|path0</td><td>RI</td></tr>
<tr><td>PB.19775.2</td><td>4-CoumarateCoA ligases(4CL)</td><td>8071_0|path0</td><td>RI</td></tr>
<tr><td>PB.20062.1</td><td>4-CoumarateCoA ligases(4CL)</td><td>7837_0|path0</td><td>RI</td></tr>
<tr><td>PB.20062.2</td><td>4-CoumarateCoA ligases(4CL)</td><td>7837_0|path0</td><td>RI</td></tr>
<tr><td>PB.20062.3</td><td>4-CoumarateCoA ligases(4CL)</td><td>7837_0|path0</td><td>RI</td></tr>
<tr><td>PB.20062.4</td><td>4-CoumarateCoA ligases(4CL)</td><td>7837_0|path0</td><td>RI</td></tr>
<tr><td>PB.20304.1</td><td>flavone 8-hydroxylase</td><td>7939_0|path0</td><td>RI,A3</td></tr>
<tr><td>PB.20304.2</td><td>flavone 8-hydroxylase</td><td>7939_0|path0</td><td>RI</td></tr>
<tr><td>PB.20304.3</td><td>flavone 8-hydroxylase</td><td>7939_0|path0</td><td>RI,A3,A5</td></tr>
<tr><td>PB.4908.1</td><td>4-CoumarateCoA ligases(4CL)</td><td>2491_0|path1</td><td>RI</td></tr>
<tr><td>PB.4908.2</td><td>4-CoumarateCoA ligases(4CL)</td><td>2491_0|path1</td><td>RI</td></tr>
<tr><td>PB.4991.1</td><td>R2R3MYB</td><td>12012_0|path0</td><td>RI</td></tr>
<tr><td>PB.4991.2</td><td>R2R3MYB</td><td>12012_0|path0</td><td>RI</td></tr>
<tr><td>PB.5982.10</td><td>phenylalanine ammonia-lyase</td><td>5440_0|path7</td><td>RI</td></tr>
<tr><td>PB.5982.12</td><td>phenylalanine ammonia-lyase</td><td>5440_0|path1</td><td>RI</td></tr>
<tr><td>PB.5982.13</td><td>phenylalanine ammonia-lyase</td><td>5440_0|path3</td><td>RI</td></tr>
<tr><td>PB.5982.14</td><td>phenylalanine ammonia-lyase</td><td>5440_0|path2</td><td>RI</td></tr>
<tr><td>PB.5982.16</td><td>phenylalanine ammonia-lyase</td><td>5440_0|path8</td><td>RI</td></tr>
<tr><td>PB.5982.17</td><td>phenylalanine ammonia-lyase</td><td>5440_0|path1</td><td>RI</td></tr>
<tr><td>PB.5982.2</td><td>phenylalanine ammonia-lyase</td><td>5440_0|path2</td><td>RI</td></tr>
<tr><td>PB.5982.3</td><td>phenylalanine ammonia-lyase</td><td>5440_0|path7</td><td>RI</td></tr>
<tr><td>PB.5982.4</td><td>phenylalanine ammonia-lyase</td><td>5440_0|path2</td><td>RI</td></tr>
<tr><td>PB.5982.5</td><td>phenylalanine ammonia-lyase</td><td>5440_0|path2</td><td>RI</td></tr>
<tr><td>PB.5982.6</td><td>phenylalanine ammonia-lyase</td><td>5440_0|path2</td><td>RI</td></tr>
<tr><td>PB.5982.7</td><td>phenylalanine ammonia-lyase</td><td>5440_0|path7</td><td>RI</td></tr>
<tr><td>PB.5982.8</td><td>phenylalanine ammonia-lyase</td><td>5440_0|path7</td><td>RI</td></tr>
<tr><td>PB.5982.9</td><td>phenylalanine ammonia-lyase</td><td>5440_0|path1</td><td>RI</td></tr>
<tr><td>PB.976.1</td><td>4-CoumarateCoA ligases(4CL)</td><td>716_0|path0</td><td>RI</td></tr>
</table>

Table S4 Specific primers flanking the AS site for PCR amplification

| Primer | Sequence | Isoforms | Amplification length（bp) |
|---|---|---|---|
| 7939-F | CAGAGGAAGATGAAGGAGTTGG | PB.20304.1 | 467 |
| 7939-R | GAGCCCTTTGGAATGGTGTA | PB.20304.2 | 530 |
| | | PB.20304.3 | 0 |
| | | PB.20665.1 | 466 |
| | | PB.20665.2 | 467 |
| | | PB.20665.3 | 457 |
| 8950-F | TATAATCATGGCGGCGAGAAA | PB.16858.12 | 526 |
| 8950-R | GGATTAGGGCAGAGTCCAATAC | PB.16858.8 | 472 |
| | | others | 668 |
| | | others | 668 |
| 4655-F | GTGCTCTCCAGAAGGAGCTC | others | 142 |
| 4655-R | CCTGAGTATCCTTGAAGAAGAGTG | PB.12545.18 | 426 |
| 5440-F | CCACAAGATTGGTGCGTTTG | PB.14211.2 | 86 |
| 5440-R | CCTGTTAGGAAACCAGTCCCAGC | others | 177 |
